# Efficacy and safety of calcitonin gene‐related peptide antagonists in migraine treatment: A meta‐analysis

**DOI:** 10.1002/brb3.2542

**Published:** 2022-03-08

**Authors:** Tingting Huang, Yang Xu, Yajie Chen, Jing Bian, Zhaohu Chu, Shoucai Zhao, Lingsong Ma

**Affiliations:** ^1^ Department of Neurology The First Affiliated Hospital of Wannan Medical Collage Wuhu Anhui China

**Keywords:** CGRP antagonists, migraine, meta‐analysis, randomized controlled trial

## Abstract

**Introduction:**

We systematically reviewed the efficacy and safety of Calcitonin Gene‐Related Peptide (CGRP) antagonists for migraine treatment.

**Methods:**

Various databases including PubMed, Embase, The Cochrane Library, Chinese National Knowledge Infrastructure (CNKI), WanFang Data were electronically searched for randomized controlled trials (RCTs) on CGRP antagonists for migraine treatment since inception to March 2021. The trials were screened for inclusion, after which the methodological quality of the included trials was assessed. Then meta‐analysis was performed using the Revman 5.3 software.

**Results:**

A total of 26 RCTs involving 21,736 patients were included. The CGRP antagonists group included 13,635 patients while the control group included 8101 patients. Meta‐analysis showed that compared to the control group, CGRP antagonists were associated with various significant effects, including the following outcome indicators: (1) number of patients with ≥50% reduction from baseline in mean monthly migraine days (RR = 1.50, 95% CI [1.39,1.62], *p* < .00001); (2) number of patients with pain free at 2 h postdose (RR = 1.98, 95% CI [1.77, 2.20], *p* < .00001), and (3) number of patients with 2–24 h sustained pain free postdose (RR = 2.18, 95% CI [1.93, 2.46], *p* < .00001). However, the number of patients with any adverse events was significantly high in the antagonists group, relative to the control group (RR = 1.08, 95% CI [1.04, 1.12], *p* < .0001).

**Conclusions:**

CGRP antagonists are significantly effective for migraine treatment; however, they are associated with various adverse events. Due to limitations with regards to quantity and quality of the included studies, the above conclusions should be verified by more high quality studies.

## Graphical abstract

CGRP antagonists can be new drugs to treat migraine patients according to their effectiveness to relieve headaches, while the safety of the drugs still need more high‐quality research to prove.

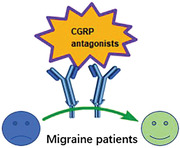

## 1 INTRODUCTION

A migraine is a chronic disabling disease, that is associated with serious harm to patients’ life and work. Chemotherapeutic options for migraine treatment are divided into two types: acute treatment and preventive treatment drugs. Acute treatment drugs include nonsteroidal anti‐inflammatory drugs (NSAIDs), triptans, and ergotamines, while preventive treatment drugs include tricyclic antidepressants (TCAs), anticonvulsants, β‐receptor blockers and calcium channel antagonists among others. The clinical pathogenesis of migraines has not been clearly established; however, various theories have been proposed to explain their development. These include the classical trigeminal neurovascular theory, in which 5‐hydroxytryptamine(5‐HT), CGRP, pituitary adenylate cyclase activating peptide all play important roles (Edvinsson et al., 2020). As new migraine treatment drugs, CGRP antagonists have been widely researched. These antagonists can be administered in both acute and interictal stages because they inhibit vasodilation and neuroinflammation (Benemei et al., 2009; Barbanti et al., 2017). CGRPs are widely expressed in multiple sites, including precranial vessels, nerve cells and neurogliocytes in the trigeminal ganglion, central end of trigeminal ganglion, brain stem, cerebellum, as well as cerebral hemisphere among others (Messlinger, 2018). There are two main types of CGRP antagonists: small molecule CGRP receptor antagonists such as telcagepant, atogepant, ubrogepant, rimegepant, olcegepant among others. The other type includes macromolecule CGRP receptor antagonists, which are also known as anti‐CGRP monoclonal antibodies (mAbs) and they include erenumab (AMG334), eptinezumab (ALD403), fremanezumab (TEV‐48125), galcanezumab (LY2951742) among others. Erenumab is a monoclonal antibody targeting CGRP receptor while the rest are monoclonal antibodies targeting CGRP (Szkutnik‐Fiedler, 2020). In this study, we reviewed the efficacies and safety of CGRP antagonists for migraine treatment.

## 2 METHODS

### 2.1 Inclusion and exclusion criteria

#### 2.1.1 Study types

RCTs on the efficacy and safety of CGRP antagonists for migraine treatment.

#### 2.1.2 Research objectives

Studies that conformed to the recommended diagnostic criteria for migraines (ICHD‐3), with or without aura, no age, sex, or course limitations.

#### 2.1.3 Intervention measures

Observation group administered with CGRP antagonists with or without conventional drugs for migraine treatment. Control group administered with conventional drugs or placebo for migraine treatment. Conventional drugs included NSAIDS, triptans, or ergotamines. There were no dosage or treatment course limitations.

#### 2.1.4 Outcome indicators

Outcome indicators are as follows: (1) number of patients with ≥50% reduction from baseline in mean monthly migraine days; (2) number of pain free patients at 2 h postdose; (3) number of patients with sustained pain free 2–24 h postdose; and (4) incidences of adverse events.

#### 2.1.5 Exclusion criteria

Exclusion criteria are as follows: (1) studies reported in non‐English language; (2) studies whose main outcome indicators were not included; (3) duplicate published studies; (4) studies limited to specific populations; and (5) unfinished research.

### 2.2 Literature retrieval strategy

Various databases including PubMed, Embase, The Cochrane Library, CNKI, WanFang Data were electronically searched from inception to March 2021 for RCTs involving CGRP antagonists for migraine treatment.

Chinese search items included migraine and CGRP antagonists, while English search items included CGRP antagonist, Calcitonin gene‐related peptide antagonist, CGRP receptor antagonist, Calcitonin gene‐related peptide receptor antagonist, eptinezumab, ALD403, fremanezumab, TEV‐48125, erenumab, AMG334, galcanezumab, LY2951742, BI 44370TA, BMS 927711, olcegepant, telcagepant, MK‐3207, rimegepant, atogepant, migraine, and randomized controlled trials.

### 2.3 Literature screening and data extraction

Two valuators (Tingting Huang and Yang Xu) independently screened the literature, extracted the data and cross‐checked it. In case of differences, an investigator (Zhaohu Chu) was consulted. Corresponding authors of the literatures were contacted when key data could not be directly obtained. Irrelevant literatures were excluded by screening the titles and summaries while valuable literatures were included by screening full articles based on inclusion and exclusion criteria. Data types that were extracted included (1) basic information of the included studies, which consisted of titles, first author name, publication journal and time; (2) basic information regarding research participants, including sample sizes, age and sex; (3) details regarding intervention drugs such as dose and course; (4) key elements to assess bias risk; and (5) outcome indicators.

### 2.4 Risk bias evaluation of the included studies

Two valuators (Tingting Huang and Yang Xu) evaluated risk bias of the included studies according to the Cochrane handbook. In case of differences, an investigator (Zhaohu Chu) was consulted.

### 2.5 Statistics analyses

Meta analyses were performed by Revman 5.3 software. Outcome indicator were enumeration data while relative risk (RR) was the effect indicator. Effect sizes are presented as point estimated value and 95% confidence interval. Heterogeneity test was performed using the chi‐square test (inspection level α = .1) as well as *I* ^2^ value. Fixed effect model was used when there were no heterogeneities among the statistical data, while the random effects model was used in case of heterogeneities.

## 3 RESULTS

### 3.1 Acquired studies

**FIGURE 1:**
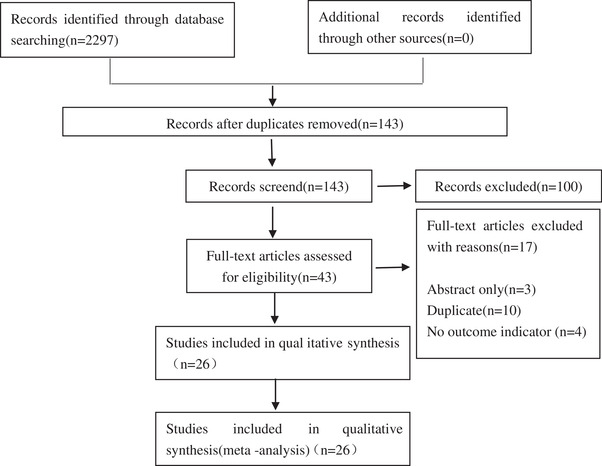
PRISMA diagram

A total of 2297 studies were acquired in the initial screening process, out of which 26 RCTs were included in the final meta‐analysis after the final screening. Fifteen RCTs evaluated small molecule CGRP receptor antagonists including BI 44370TA, BMS 927711, olcegepant (BIBN4096BS), ubrogepant (MK‐1602), telcagepant (MK‐0974), MK‐3207, rimegepant, and atogepant. Eleven RCTs evaluated anti‐CGRP monoclonal antibodies including eptinezumab (ALD403), fremanezumab (TEV‐48125), erenumab (AMG334), and galcanezumab (LY2951742). Quality evaluation of the included literatures was performed by using a flowchart based on the Preferred Reporting Items for Systematic Reviews and Meta‐Analyses (PRISMA) statement (Moher et al., 2009) (Figure 1). The Jadad scale (Jadad et al., 1996) was performed to evaluate the methodological quality of the included studies in the meta‐analysis. Each study was scored from 0 to 5 (0 represents “pool” quality and 5 represents “good” quality) according to the following judgment criteria: (1) the study was described as randomized or not, (2) the study was described as double blind or not, (3) there was a description of withdrawals and dropouts or not, (4) the method of randomization was described and appropriate or not, (5) the blinding was described and appropriate or not. A single point was given if the answer to one of the above five questions was “yes” and zero point was given if the answer was “no.” Among the included 26 RCTs, only 7 RCTs (Dodick, Lipton, Ailani et al., 2019; Hewitt, Martin et al., 2011; Lipton, Croop et al., 2019; Lipton, Dodick et al., 2019; Olesen et al., 2004; Silberstein et al., 2017; Stauffer et al., 2018) didn't get “5” scores. Besides, most RCTs got scores “≥4” scores except 1 RCT (“2” scores, Lipton, Croop et al., 2019), which illustrated that the quality of the included RCTs was relatively high.

### 3.2 Basic characteristics of the included studies and risk of bias evaluation result

The basic characteristics of the included studies are shown in Table 1 and risk of bias evaluation results are shown in Table 2. The Jadad Scale was shown in Table 3. The outcome indicators of the included studies are shown in Table 4.

**TABLE 1:** Basic characteristics of included studies

Included studies	Research type	Follow‐up time	Number of cases (intervening measure)	Mean age (mean value ± SD/median)	Sex (F/M)
Dodick et al., 2018	RCT Phase III NCT02483585	12 weeks	286 (Erenumab 70 mg) 291 (Placebo)	42 ± 11 42 ± 12	245/41 247/44
Diener et al., 2010	RCT Phase II NCT00751803	1 week	64 (BI44370TA 50 mg) 65 (BI44370TA 200 mg) 73 (BI44370TA 400 mg) 69 (Eletriptan 40 mg) 70 (Placebo)	42.8 ± 11.7 41.2 ± 9.7 41.1 ± 10.0 37.9 ± 10.1 38.2 ± 10.3	54/10 53/12 55/18 61/8 61/9
Marcus et al., 2014	RCT Phase II NCT01430442	11 weeks	85 (BMS‐927711 10 mg) 68 (BMS‐927711 25 mg) 91 (BMS‐927711 75 mg) 90 (BMS‐927711 150 mg) 121 (BMS‐927711 300 mg) 92 (BMS‐927711 600 mg) 109 (Sumatriptan 100 mg) 229 (Placebo)	41.1 ± 10.36 36.5 ± 11.92 38.5 ± 11.87 39.2 ± 11.26 41.9 ± 11.46 39.3 ± 13.01 40.6 ± 10.47 37.9 ± 11.36	67/18 61/7 81/10 63/27 101/20 76/16 91/18 196/33
Olesen et al., 2004	RCT	24 h	1 (BIBN4096BS 0.25 mg) 4 (BIBN4096BS 0.5 mg) 20 (BIBN4096BS 1 mg) 32 (BIBN4096BS 2.5 mg) 16 (BIBN4096BS 5 mg) 12 (BIBN4096BS 10 mg) 41 (Placebo)	43 42 49 45 52 47 47	1/0 3/1 18/2 29/3 13/3 7/5 29/12
Goadsby et al., 2017	RCT NCT02456740	6 months	317 (Erenumab 70 mg) 319 (Erenumab 140 mg) 319 (Placebo)	41.1 ± 11.3 40.4 ± 11.1 41.3 ± 11.2	268/49 272/47 274/45
Reuter et al., 2018	RCT Phase IIIb NCT03096834	12 weeks	121 (Erenumab 140 mg) 125 (Placebo)	44.6 ± 10.5 44.2 ± 10.6	97/24 103/22
Dodick, Lipton, Ailani et al., 2019	RCT Phase IIb NCT02275117	49 weeks	130 (Eptinezumab 10 mg) 122 (Eptinezumab 30 mg) 122 (Eptinezumab 100 mg) 121 (Eptinezumab 300 mg) 121 (Placebo)	36.4 ± 10.3 35.7 ± 9.4 36.7 ± 9.4 37.2 ± 10.0 37.2 ± 9.2	113/27 111/9 104/18 98/23 109/12
Tepper et al., 2017	RCT Phase II NCT02066415	12 weeks	191 (Erenumab 70 mg) 190 (Erenumab 140 mg) 286 (Placebo)	41.4 ± 11.3 42.9 ± 11.1 42.1 ± 11.3	166/25 160/30 226/60
Silberstein et al., 2017	RCT Phase II NCT02621931	16 weeks	376 (Fremanezumab quarterly) 379 (Fremanezumab monthly) 375 (Placebo)	42.0 ± 12.4 40.6 ± 12.0 41.4 ± 12.0	331/45 330/49 330/45
Voss et al., 2016	RCT Phase IIb NCT01613248	48 h	107 (Ubrogepant 1 mg) 108 (Ubrogepant 10 mg) 104 (Ubrogepant 25 mg) 106 (Ubrogepant 50 mg) 102 (Ubrogepant 100 mg) 113 (Placebo)	39.6 ± 10.7 41.1 ± 10.9 41.4 ± 11.5 40.7 ± 12.3 41.9 ± 11.0 40.5 ± 11.7	95/12 92/16 91/13 92/14 90/12 99/14
Hewitt, Aurora et al., 2011	RCT NCT00758836	5 days	145 (Telcagepant +Ibuprofen) 133 (Telcagepant +Acetaminophen) 138 (Telcagepant) 147 (Placebo)	39.2 ± 11.7 42.3 ± 12.7 39.3 ± 11.6 41.9 ± 12.0	123/22 118/15 119/19 130/17
Ho et al., 2008	RCT Phase II	14 days	14 (MK‐0974 25 mg) 15 (MK‐0974 50 mg) 16 (MK‐0974 100 mg) 12 (MK‐0974 200 mg) 39 (MK‐0974 300 mg) 45 (MK‐0974 400 mg) 40 (MK‐0974 600 mg) 34 (Rizatriptan 10 mg) 115 (Placebo)	43 41.5 40.9 34.3 40.5 40.1 44.5 40.2 42.2	11/3 14/1 14/2 9/3 34/5 42/3 36/4 28/6 104/11
Hewitt, Martin et al., 2011	RCT Phase II NCT00712725	14 days	33 (MK‐3207 2.5 mg) 47 (MK‐3207 5 mg) 67 (MK‐3207 10 mg) 67 (MK‐3207 20 mg) 68 (MK‐3207 50 mg) 62 (MK‐3207 100 mg) 63 (MK‐3207 200 mg) 140 (Placebo)	43.3 ± 10.5 43.4 ± 11.1 44.1 ± 10.0 44.1 ± 11.3 42.2 ± 10.8 42.4 ± 10.9 40.5 ± 10.7 42.1 ± 11.2	27/6 40/7 62/5 54/13 62/6 52/10 54/9 125/15
Ho et al., 2010	RCT Phase III NCT00483704	14 days	573 (Telcagepant 140 mg) 549 (Telcagepant 280 mg) 555 (Placebo)	43.4 ± 11.7 42.4 ± 11.5 42.5 ± 11.6	490/83 471/78 463/92
Connor et al., 2009	RCT Phase III NCT00432237	14 days	177 (Telcagepant 50 mg) 381 (Telcagepant 150 mg) 371 (Telcagepant 300 mg) 365 (placebo)	41.4 ± 11.3 41.6 ± 11.0 41.8 ± 11.6 41.9 ± 11.9	156/21 329/52 320/51 318/47
Sun et al., 2016	RCT Phase II NCT01952574	256 weeks	108 (AMG 334 7 mg) 108 (AMG 334 21 mg) 107 (AMG 334 70 mg) 160 (placebo)	40.3 ± 10.9 39.9 ± 12.3 42.6 ± 9.9 41.4 ± 10.0	88/20 87/21 82/25 132/28
Dodick, Lipton, Silberstein et al., 2019	RCT NCT02828020	24 h	466 (Ubrogepant 50 mg) 485 (Ubrogepant 100 mg) 485 (Placebo)	40.1 ± 11.7 40.6 ± 12.0 40.9 ± 11.7	418/48 418/67 430/55
Lipton, Croop et al., 2019	RCT NCT03235479	8 h	582 (Rimegepant) 580 (Placebo)	N/A N/A	N/A N/A
Lipton, Dodick et al., 2019	RCT Phase III NCT03237845	7 days	537 (Rimegepant) 535 (Placebo)	40.2 ± 11.9 40.9 ± 12.1	479/58 472/63
Lipton et al., 2018	RCT Phase III NCT02867709	48 h	478 (Ubrogepant 25 mg) 488 (Ubrogepant 50 mg) 499 (Placebo)	41.6 ± 12.4 41.2 ± 12.5 41.7 ± 12.1	431/47 444/44 442/57
Croop et al., 2019	RCT Phase III NCT03461757	80 days	669 (Rimegepant 75 mg) 682 (Placebo)	40.3 ± 12.1 40.0 ± 11.9	568/101 579/103
Goadsby et al., 2019	RCT Phase IIb/III NCT02848326	12 weeks	93 (Atogepant 10 mg QD) 183 (Atogepant 30 mg QD) 86 (Atogepant 30 mg BID) 186 (Atogepant 60 mg QD) 91 (Atogepant 60 mg BID) 186 (Placebo)	39.4 ± 12.4 41.0 ± 13.6 38.5 ± 11.2 40.4 ± 11.7 39.7 ± 11.9 40.5 ± 11.7	82/11 166/17 73/13 156/30 83/8 154/32
Skljarevski et al., 2018	RCT Phase III NCT02614196	6 months	231 (Galcanezumab120 mg) 223 (Galcanezumab240 mg) 461 (Placebo)	40.9 ± 11.2 41.9 ± 10.8 42.3 ± 11.3	197/34 191/32 393/68
Detke et al., 2018	RCT Phase III NCT02614261	3 months	278 (Galcanezumab120 mg) 277 (Galcanezumab240 mg) 558 (Placebo)	39.7 ± 11.9 41.1 ± 12.4 41.6 ± 12.1	237/41 226/51 483/75
Stauffer et al., 2018	RCT Phase III NCT02614183	6 months	213 (Galcanezumab120 mg) 212 (Galcanezumab240 mg) 433 (Placebo)	40.9 ± 11.9 39.1 ± 11.5 41.3 ± 11.4	181/97 175/102 362/196
Mulleners et al., 2020	RCT Phase IIIb NCT03559257	3 months	232 (Galcanezumab120 mg) 230 (Placebo)	45.9 ± 11.3 45.7 ± 12.3	195/37 202/28

**TABLE 2:** Risk of bias evaluation result

Included studies	Random method	Blind method	Distribution of hidden	Data integrity	Selective report data
Dodick et al., 2018	Computer randomness	Double blind	Interactive voice response system	Integrity	No
Diener et al., 2010	Computer randomness	Double blind	Unclear	Integrity	No
Marcus et al., 2014	Interactive voice response system	Double blind	Unclear	Integrity	No
Olesen et al., 2004	Unclear	Double blind	Unclear	Integrity	No
Goadsby et al., 2017	Interactive voice/web response system	Double blind	Unclear	Integrity	No
Reuter et al., 2018	Interactive voice response system	Double blind	unclear	Integrity	No
Dodick, Lipton, Ailani et al., 2019	Interactive web response system	Double blind	Unclear	Integrity	No
Tepper et al., 2017	Interactive voice/web response system	Double blind	Unclear	Integrity	No
Silberstein et al., 2017	Electronic interactive response system	Double blind	Unclear	Integrity	No
Voss et al., 2016	Computer randomness	Double blind	Unclear	Integrity	No
Hewitt, Aurora et al., 2011	Computer randomness	Double blind	Unclear	Integrity	No
Ho et al., 2008	Computer randomness	Double blind	Unclear	Integrity	No
Hewitt, Martin et al., 2011	Computer randomness	Double blind	Interactive voice response system	Integrity	No
Ho et al., 2010	Computer randomness	Double blind	Central interactive voice system	Integrity	No
Connor et al., 2009	Computer randomness	Double blind	Central interactive voice system	Integrity	No
Sun et al., 2016	Interactive voice/web response system	Double blind	Central distribution	Integrity	No
Dodick, Lipton, Silberstein et al., 2019	Automated network response system	Double blind	Unclear	Integrity	No
Lipton, Croop et al., 2019	unclear	Double blind	Unclear	Integrity	No
Lipton, Dodick et al., 2019	Interactive web response system	Double blind	Unclear	Integrity	No
Lipton et al., 2018	Computer randomness	Double blind	Interactive web response system	Integrity	No
Croop et al., 2019	Interactive web response system	Double blind	Interactive web response system	Integrity	No
Goadsby et al., 2019	Interactive web response system	Double blind	Unclear	Integrity	No
Skljarevski et al., 2018	Computer randomness	Double blind	Interactive web response system	Integrity	No
Detke et al., 2018	Interactive web response system	Double blind	Unclear	Integrity	No
Stauffer et al., 2018	Computer randomness	Double blind	Interactive web response system	Integrity	No
Mulleners et al., 2020	Computer randomness	Double blind	Interactive web response system	Integrity	No

**TABLE 3:** Jadad Scale

Included studies	Randomized	Double blinded	Description of withdrawals and drop outs	Randomization Method described and appropriate	Blinding method described and appropriate	Total
Dodick et al., 2018	1	1	1	1	1	5
Diener et al., 2010	1	1	1	1	1	5
Marcus et al., 2014	1	1	1	1	1	5
Olesen et al., 2004	1	1	1	1	0	4
Goadsby et al., 2017	1	1	1	1	1	5
Reuter et al., 2018	1	1	1	1	1	5
Dodick, Lipton, Ailani et al., 2019	1	1	1	1	0	4
Tepper et al., 2017	1	1	1	1	1	5
Silberstein et al., 2017	1	1	1	1	0	4
Voss et al., 2016	1	1	1	1	1	5
Hewitt, Aurora et al., 2011	1	1	1	1	1	5
Ho et al., 2008	1	1	1	1	1	5
Hewitt, Martin et al., 2011	1	1	1	1	0	4
Ho et al., 2010	1	1	1	1	1	5
Connor et al., 2009	1	1	1	1	1	5
Sun et al., 2016	1	1	1	1	1	5
Dodick, Lipton, Silberstein et al., 2019	1	1	1	1	1	5
Lipton, Croop et al., 2019	1	1	0	0	0	2
Lipton, Dodick et al., 2019	1	1	1	1	0	4
Lipton et al., 2018	1	1	1	1	1	5
Croop et al., 2019	1	1	1	1	1	5
Goadsby et al., 2019	1	1	1	1	1	5
Skljarevski et al., 2018	1	1	1	1	1	5
Detke et al., 2018	1	1	1	1	1	5
Stauffer et al., 2018	1	1	1	1	0	4
Mulleners et al., 2020	1	1	1	1	1	5

**TABLE 4:** The outcome indicators of the included studies

				Heterogeneity		Meta‐analysis	
Outcome indicator	Intervening measure	Study numbers included	Case numbers (T/C)	*p*	*I* ^2^	RR(95%CI)	*p*
Number of patients with ≥50% reduction from baseline in mean monthly migraine day	Erenumab 7 mg vs. Placebo	1 (Sun et al., 2016)	104/144	‐	‐	0.97(0.65,1.43)	.86
	Erenumab 21 mg vs. Placebo	1 (Sun et al., 2016)	93/144	‐	‐	1.15(0.79,1.68)	.46
	Erenumab 70 mg vs. Placebo	3 (Dodick et al., 2018; Goadsby et al., 2017; Sun et al., 2016)	693/748	.49	0%	1.50(1.30,1.73)	<.00001
	Erenumab 140 mg vs. Placebo	2 (Goadsby et al., 2017; Reuter et al., 2018)	437/440	.58	0%	1.92(1.58,2.35)	<.00001
	Atogepant 10 mgQd vs. Placebo	1 (Goadsby et al., 2019)	92/178	‐	‐	1.42(1.11,1.83)	.006
	Atogepant 30 mgQd vs. Placebo	1 (Goadsby et al., 2019)	182/178	‐	‐	1.32(1.05,1.65)	.02
	Atogepant 60 mgQd vs. Placebo	1 (Goadsby et al., 2019)	177/178	‐	‐	1.28(1.02,1.61)	.03
	Atogepant 30 mgBid vs. Placebo	1 (Goadsby et al., 2019)	79/178	‐	‐	1.44(1.11.1.86)	.006
	Atogepant 60 mgBid vs. Placebo	1 (Goadsby et al., 2019)	87/178	‐	‐	1.53(1.20,1.96)	.0005
	Eptinezumab 10 mg vs. Placebo	1 (Dodick, Lipton, Ailani et al., 2019)	123/116	‐	‐	1.08(0.80,1.46)	.60
	Eptinezumab 30 mg vs. Placebo	1 ( Dodick, Lipton, Ailani et al., 2019)	117/116	‐	‐	1.37(1.04,0.80)	.02
	Eptinezumab 100 mg vs. Placebo	1 ( Dodick, Lipton, Ailani et al., 2019)	118/116	‐	‐	1.36(1.03,1.79)	.03
	Eptinezumab 300 mg vs. Placebo	1 (Dodick, Lipton, Ailani et al., 2019)	114/116	‐	‐	1.41(1.07,0.85)	.01
	Galcanezumab 120 mg vs. Placebo	3 (Detke et al., 2018; Mulleners et al., 2020; Skljarevski et al., 2018)	736/1229	.03	71%	1.94(1.47,2.56)	<.00001
	Galcanezumab 240 mg vs. Placebo	2 (Detke et al., 2018; Skljarevski et al., 2018)	497/999	.45	0%	1.62(1.41,1.87)	<.00001
	Total	9 (Detke et al., 2018; Dodick et al., 2018; Dodick, Lipton, Ailani et al., 2019; Goadsby et al., 2017; Goadsby et al., 2019; Mulleners et al., 2020; Reuter et al., 2018; Skljarevski et al., 2018; Sun et al., 2016)	3649/5058	.008	46%	1.50(1.39,1.62)	<.00001
Number of pain free patients at 2 h postdose	BI44370TA 50 mg vs. Placebo	1 (Diener et al., 2010)	64/70	‐	‐	0.91(0.29,2.84)	.87
	BI44370TA 200 mg vs. Placebo	1 (Diener et al., 2010)	65/70	‐	‐	2.51(1.03,6.15)	.04
	BI44370TA 400 mg vs. Placebo	1 (Diener et al., 2010)	73/70	‐	‐	3.20(1.36,7.49)	.007
	BIBN4096BS 1 mg vs. Placebo	1 (Olesen et al., 2004)	20/41	‐	‐	8.2(0.98,68.66)	.05
	BIBN4096BS 2.5 mg vs. Placebo	1 (Olesen et al., 2004)	32/41	‐	‐	17.94(2.49,129.32)	.004
	BIBN4096BS 5 mg vs. Placebo	1 (Olesen et al., 2004)	16/41	‐	‐	10.25(1.24,84.86)	.03
	BIBN4096BS 10 mg vs. Placebo	1 (Olesen et al., 2004)	12/41	‐	‐	10.25(1.17,89.76)	.04
	Ubrogepant1 mg vs. Placebo	1 (Voss et al., 2016)	107/112	‐		0.63(0.24,1.67)	.35
	Ubrogepant 10 mg vs. Placebo	1 (Voss et al., 2016)	108/112	‐	‐	1.66(0.79,3.49)	.18
	Ubrogepant 25 mg vs. Placebo	2 (Lipton et al., 2018; Voss et al., 2016)	538/568	.19	41%	1.68(1.08,2.62)	.02
	Ubrogepant 50 mg vs. Placebo	3 (Dodick, Lipton, Silberstein et al., 2019; Lipton et al., 2018; Voss et al., 2016)	991/1024	.54	0%	1.62(1.32,1.99)	<.00001
	Ubrogepant 100 mg vs. Placebo	2 (Dodick, Lipton, Silberstein et al., 2019; Voss et al., 2016)	550/568	.22	34%	2.04(1.35,3.08)	.0007
	Telcagepant25 mg vs. Placebo	1 (Ho et al., 2008)	14/115	‐	‐	1.54(0.51,4.63)	.44
	Telcagepant 50 mg vs. Placebo	2 (Connor et al., 2009; Ho et al., 2008)	192/480	.06	72%	2.16(0.99,4.73)	.05
	Telcagepant 100 mg vs. Placebo	1 (Ho et al., 2008)	16/115	‐	‐	1.35(0.44,4.12)	.60
	Telcagepan 140 mg vs. Placebo	1 (Ho et al., 2010)	556/539	‐	‐	2.15(1.60,2.89)	<.00001
	Telcagepant 150 mg vs. Placebo	1 (Connor et al., 2009)	381/365	‐	‐	2.16(1.53,3.06)	<.0001
	Telcagepant 200 mg vs. Placebo	1 (Ho et al., 2008)	12/115	‐	‐	1.20(0.31,4.59)	.79
	Telcagepant 280 mg vs. Placebo	2 ( Hewitt, Aurora et al., 2011; Ho et al., 2010)	704/710	.64	0%	2.55(1.99,3.27)	<.00001
	Telcagepant 300 mg vs. Placebo	2 (Connor et al., 2009; Ho et al., 2008)	409/480	.27	16%	2.49(1.77,3.49)	<.00001
	Telcagepant 400 mg vs. Placebo	1 (Ho et al., 2008)	45/115	‐	‐	1.76(0.88,3.49)	.11
	Telcagepant 600 mg vs. Placebo	1 (Ho et al., 2008)	40/115	‐	‐	2.34(1.24,4.42)	.009
	Rimegepant 75 mg vs. Placebo	3 (Croop et al., 2019; Lipton, Croop et al., 2019; Lipton, Dodick et al., 2019)	1749/1758	.15	48%	1.63(1.31,2.03)	<.0001
	MK‐3207 2.5 mg vs. Placebo	1 (Hewitt, Martin et al., 2011)	32/133	‐	‐	1.28(0.45,3.66)	.65
	MK‐3207 5 mg vs. Placebo	1 (Hewitt, Martin et al., 2011)	44/133			1.16(0.44,3.08)	.76
	MK‐3207 10 mg vs. Placebo	1 (Hewitt, Martin et al., 2011)	63/133			2.60(1.33,5.07)	.005
	MK‐3207 20 mg vs. Placebo	1 (Hewitt, Martin et al., 2011)	63/133	‐	‐	1.95(0.94,4.02)	.07
	MK‐3207 50 mg vs. Placebo	1 (Hewitt, Martin et al., 2011)	65/133	‐	‐	2.20(1.10,4.41)	.03
	MK‐3207 100 mg vs. Placebo	1 (Hewitt, Martin et al., 2011)	59/133	‐	‐	2.43(1.22,4.84)	.01
	MK‐3207 200 mg vs. Placebo	1 (Hewitt, Martin et al., 2011)	58/133	‐	‐	3.70(1.99,6.88)	<.0001
	Total	13 (Diener et al., 2010; Olesen et al., 2004; Voss et al., 2016; Lipton et al., 2018; Dodick, Lipton, Silberstein et al., 2019; Ho et al., 2008; Connor et al., 2009; Ho et al., 2010; Croop et al., 2019; Lipton, Croop et al., 2019; Lipton, Dodick et al., 2019; Hewitt, Aurora et al., 2011; Hewitt, Martin et al., 2011)	7078/8596	.02	36%	1.98(1.77,2.20)	<.00001
Number of patients with 2–24 h sustained pain free postdose	BI44370TA 50 mg vs. Placebo	1 (Diener et al., 2010)	64/70	‐	‐	0.66(0.16,2.64)	.55
	BI44370TA 200 mg vs. Placebo	1 (Diener et al., 2010)	65/70	‐	‐	2.80(1.06,7.42)	.04
	BI44370TA 400 mg vs. Placebo	1 (Diener et al., 2010)	73/70	‐	‐	2.88(1.10,7.49)	.03
	BMS‐927711 10 mg vs. Placebo	1 (Marcus et al., 2014)	71/203	‐	‐	1.72(0.79,3,75)	.18
	BMS‐927711 25 mg vs. Placebo	1 (Marcus et al., 2014)	61/203	‐	‐	2.22(1.05,4.68)	.04
	BMS‐927711 75 mg vs. Placebo	1 (Marcus et al., 2014)	86/203	‐	‐	3.78(2.09,6.84)	<.0001
	BMS‐927711 150 mg vs. Placebo	1 (Marcus et al., 2014)	85/203	‐	‐	3.82(2.11,6.92)	<.00001
	BMS‐927711 300 mg vs. Placebo	1 (Marcus et al., 2014)	111/203	‐	‐	3.54(1.98,6.31)	<.0001
	BMS‐927711 600 mg vs. Placebo	1 (Marcus et al., 2014)	82/203	‐	‐	2.81(1.47,5.35)	.002
	Ubrogepant 1 mg vs. Placebo	1 (Voss et al., 2016)	107/113	‐	‐	0.75(0.25,2.30)	.62
	Ubrogepant 10 mg vs. Placebo	1 (Voss et al., 2016)	108/113	‐	‐	1.49(0.59,3.79)	.40
	Ubrogepant 25 mg vs. Placebo	2 (Lipton et al., 2018; Voss et al., 2016)	538/569	.39	0%	1.68(1.17,2.40)	.004
	Ubrogepant 50 mg vs. Placebo	3 (Voss et al., 2016; Lipton et al., 2018; Dodick, Lipton, Silberstein et al., 2019)	988/1021	.61	0%	1.66(1.28,2.16)	.0001
	Ubrogepant 100 mg vs. Placebo	2 (Voss et al., 2016; Dodick, Lipton, Silberstein et al., 2019)	543/565	.17	46%	2.18(1.24,3.86)	.007
	Telcagepant 25 mg vs. Placebo	1 (Ho et al., 2008)	14/115	‐	‐	0.63(0.09,4.47)	.65
	Telcagepant 50 mg vs. Placebo	2 (Ho et al., 2008; Connor et al., 2009)	192/480	.10	63%	2.73(1.31,5.70)	.008
	Telcagepant 100 mg vs. Placebo	1 (Ho et al., 2008)	16/115	‐	‐	1.66(0.53,5.19)	.38
	Telcagepant 140 mg vs. Placebo	1 (Ho et al., 2010)	553/537	‐	‐	2.11(1.44,3.09)	.0001
	Telcagepant 150 mg vs. Placebo	1 (Connor et al., 2009)	381/365	‐	‐	2.28(1.48,3.53)	.0002
	Telcagepant 200 mg vs. Placebo	1 (Ho et al., 2008)	12/115	‐	‐	0.74(0.11,5.16)	.76
	Telcagepant 280 mg vs. Placebo	1 (Ho et al., 2010)	531/537	‐	‐	2.63(1.81,3.81)	<.00001
	Telcagepant 300 mg vs. Placebo	2 (Ho et al., 2008; Connor et al., 2009)	409/480	.35	0%	2.71(1.89,3.88)	<.00001
	Telcagepant 400 mg vs. Placebo	1 (Ho et al., 2008)	45/115	‐	‐	1.97(0.93,4.16)	.08
	Telcagepant 600 mg vs. Placebo	1 (Ho et al., 2008)	40/115	‐	‐	2.88(1.46,5.67)	.002
	Rimegepant 75 mg vs. Placebo	1 (Croop et al., 2019)	669/682	‐	‐	1.52(1.24,1.87)	<.0001
	MK‐3207 2.5 mg vs. Placebo	1 (Hewitt, Martin et al., 2011)	32/133	‐	‐	1.66(0.56,4.96)	.36
	MK‐3207 5 mg vs. Placebo	1 (Hewitt, Martin et al., 2011)	44/133	‐	‐	0.60(0.14,2.65)	.50
	MK‐3207 10 mg vs. Placebo	1 (Hewitt, Martin et al., 2011)	63/133	‐	‐	2.74(1.27,5.92)	.010
	MK‐3207 20 mg vs. Placebo	1 (Hewitt, Martin et al., 2011)	63/133	‐	‐	2.11(0.93,4.81)	.08
	MK‐3207 50 mg vs. Placebo	1 (Hewitt, Martin et al., 2011)	65/133	‐	‐	2.46(1.12,5.38)	.02
	MK‐3207 100 mg vs. Placebo	1 (Hewitt, Martin et al., 2011)	59/133	‐	‐	2.71(1.24,5.91)	.01
	MK‐3207 200 mg vs. Placebo	1 (Hewitt, Martin et al., 2011)	58/133	‐	‐	3.90(1.90,7.99)	.0002
	Total	10 (Diener et al., 2010; Marcus et al., 2014; Voss et al., 2016; Lipton et al., 2018; Dodick, Lipton, Silberstein et al., 2019; Ho et al., 2008; Ho et al., 2010; Connor et al., 2009; Croop et al., 2019; Hewitt, Martin et al., 2011)	6228/8396	.03	32%	2.18(1.93,2.46)	<.00001
Adverse events	Erenumab 7 mg vs. Placebo	1 (Sun et al., 2016)	108/153	‐	‐	0.93(0.73,1.19)	.57
	Erenumab 21 mg vs. Placebo	1 (Sun et al., 2016)	105/153	‐	‐	0.96(0.76,1.22)	.73
	Erenumab 70 mg vs. Placebo	3 (Dodick et al., 2018; Goadsby et al., 2017; Sun et al., 2016)	703/761	.65	0%	0.91(0.83,1.00)	.05
	Erenumab 140 mg vs. Placebo	2 (Goadsby et al., 2017; Reuter et al., 2018)	438/443	.31	5%	0.91(0.81,1.03)	.12
	Telcagepant 25 mg vs. Placebo	1 (Ho et al., 2008)	17/47	‐	‐	0.65(0.25,1.66)	.37
	Telcagepant 50 mg vs. Placebo	2 (Ho et al., 2008; Connor et al., 2009)	196/413	.50	0%	1.08(0.85,1.38)	.53
	Telcagepant 100 mg vs. Placebo	1 (Ho et al., 2008)	27/47	‐	‐	0.41(0.15,1.09)	.07
	Telcagepant 140 mg vs. Placebo	1 (Ho et al., 2010)	573/561	‐	‐	1.17(1.02,1.34)	.03
	Telcagepant 150 mg vs. Placebo	1 (Connor et al., 2009)	381/366	‐	‐	0.99(0.80,1.23)	.96
	Telcagepant 200 mg vs. Placebo	1 (Ho et al., 2008)	18/47	‐	‐	0.92(0.43,1.96)	.83
	Telcagepant 280 mg vs. Placebo	2 (Ho et al., 2010; Hewitt, Aurora et al., 2011)	713/732	.38	0%	1.15(1.00,1.31)	.05
	Telcagepant 300 mg vs. Placebo	2 (Ho et al., 2008; Connor et al., 2009)	421/413	.63	0%	1.10(0.91,1.33)	.33
	Telcagepant 400 mg vs. Placebo	1 (Ho et al., 2008)	52/47	‐	‐	1.01(0.60,1.70)	.97
	Telcagepant 600 mg vs. Placebo	1 (Ho et al., 2008)	49/47	‐	‐	1.13(0.68,1.88)	.64
	Atogepant 10 mg Qd vs. Placebo	1 (Goadsby et al., 2019)	93/186	‐	‐	1.33(1.08,1.63)	.007
	Atogepant 30 mg Qd vs. Placebo	1 (Goadsby et al., 2019)	183/186	‐	‐	1.27(1.06,1.53)	.01
	Atogepant 60 mg Qd vs. Placebo	1 (Goadsby et al., 2019)	186/186	‐	‐	1.16(0.96,1.41)	.12
	Atogepant 30 mg Bid vs. Placebo	1 (Goadsby et al., 2019)	86/186	‐	‐	1.22(0.98,1.53)	.08
	Atogepant 60 mg Bid vs. Placebo	1 (Goadsby et al., 2019)	91/186	‐	‐	1.18(0.94,1.48)	.16
	Eptinezumab 10 mg vs. Placebo	1 ( Dodick, Lipton, Ailani et al., 2019)	130/121	‐	‐	1.01(0.82,1.26)	.91
	Eptinezumab 30 mg vs. Placebo	1 ( Dodick, Lipton, Ailani et al., 2019)	122/121	‐	‐	0.82(0.64,1.05)	.11
	Eptinezumab 100 mg vs. Placebo	1 ( Dodick, Lipton, Ailani et al., 2019)	122/121	‐	‐	1.02(0.82,1.27)	.85
	Eptinezumab 300 mg vs. Placebo	1 ( Dodick, Lipton, Ailani et al., 2019)	121/121	‐	‐	1.13(0.92,1.39)	.24
	BI44370TA 50 mg vs. Placebo	1 (Diener et al., 2010)	64/70	‐	‐	1.88(0.79,4.47)	.16
	BI44370TA 200 mg vs. Placebo	1 (Diener et al., 2010)	65/70	‐	‐	0.62(0.19,2.01)	.42
	BI44370TA 400 mg vs. Placebo	1 (Diener et al., 2010)	73/70	‐	‐	0.96(0.35,2.59)	.93
	Ubrogepant 1 mg vs. Placebo	1 (Voss et al., 2016)	107/113	‐	‐	1.24(0.81,1.91)	.32
	Ubrogepant 10 mg vs. Placebo	1 (Voss et al., 2016)	108/113	‐	‐	1.08(0.69,1.69)	.72
	Ubrogepant 25 mg vs. Placebo	1 (Voss et al., 2016)	103/113	‐	‐	0.82(0.50,1.36)	.44
	Ubrogepant 50 mg vs. Placebo	2 (Voss et al., 2016; Dodick, Lipton, Silberstein et al., 2019)	573/598	.60	0%	0.78(0.59,1.05)	.10
	Ubrogepant 100 mg vs. Placebo	2 (Voss et al., 2016; Dodick, Lipton, Silberstein et al., 2019)	587/598	.80	0%	1.24(0.97,1.60)	.09
	Fremanezumab quarterly vs. Placebo	1 (Silberstein et al., 2017)	376/375	‐	‐	1.10(1.00,1.22)	.06
	Fremanezumab monthly vs. Placebo	1 (Silberstein et al., 2017)	379/375	‐	‐	1.11(1.01,1.23)	.03
	MK‐3207 2.5 mg vs. Placebo	1 (Hewitt, Martin et al., 2011)	32/142	‐	‐	1.53(0.83,2.81)	.17
	MK‐3207 5 mg vs. Placebo	1 (Hewitt, Martin et al., 2011)	47/142	‐	‐	1.88(1.15,3.05)	.01
	MK‐3207 10 mg vs. Placebo	1 (Hewitt, Martin et al., 2011)	66/142	‐	‐	1.26(0.75,2.13)	.38
	MK‐3207 20 mg vs. Placebo	1 (Hewitt, Martin et al., 2011)	67/142	‐	‐	1.32(0.79,2.19)	.29
	MK‐3207 50 mg vs. Placebo	1 (Hewitt, Martin et al., 2011)	68/142	‐	‐	1.30(0.78,2.16)	.32
	MK‐3207 100 mg vs. Placebo	1 (Hewitt, Martin et al., 2011)	62/142	‐	‐	1.50(0.91,2.46)	.11
	MK‐3207 200 mg vs. Placebo	1 (Hewitt, Martin et al., 2011)	63/142	‐	‐	1.32(0.79,2.22)	.29
	Rimegepant 75 mg vs. Placebo	2 (Croop et al., 2019; Lipton, Dodick et al., 2019)	1225/1236	.86	0%	1.23(1.01,1.50)	.04
	Galcanezumab 120 mg vs. Placebo	4 (Detke et al., 2018; Mulleners et al., 2020; Skljarevski et al., 2018; Stauffer et al., 2018)	937/1681(	.38	4%	1.07(1.00,1.15)	.04
	Galcanezumab 240 mg vs. Placebo	3 (Detke et al., 2018; Skljarevski et al., 2018; Stauffer et al., 2018)	730/1451	.96	0%	1.14(1.06,1.22)	.0003
	Total	21 (Sun et al., 2016; Dodick et al., 2018; Goadsby et al., 2017; Reuter et al., 2018; Ho et al., 2008; Connor et al., 2009; Ho et al., 2010; Hewitt, Aurora et al., 2011; Goadsby et al., 2019; Dodick, Lipton, Ailani et al., 2019; Diener et al., 2010; Voss et al., 2016; Dodick, Lipton, Silberstein et al., 2019; Silberstein et al., 2017; Hewitt, Martin et al., 2011; Croop et al., 2019; Lipton, Dodick et al., 2019; Detke et al., 2018; Mulleners et al., 2020; Skljarevski et al., 2018; Stauffer et al., 2018)	10667/13501	.005	39.8%	1.08(1.04,1.12)	<.0001

### 3.3 Meta‐analysis results

#### 3.3.1 Efficacy

**FIGURE 2:**
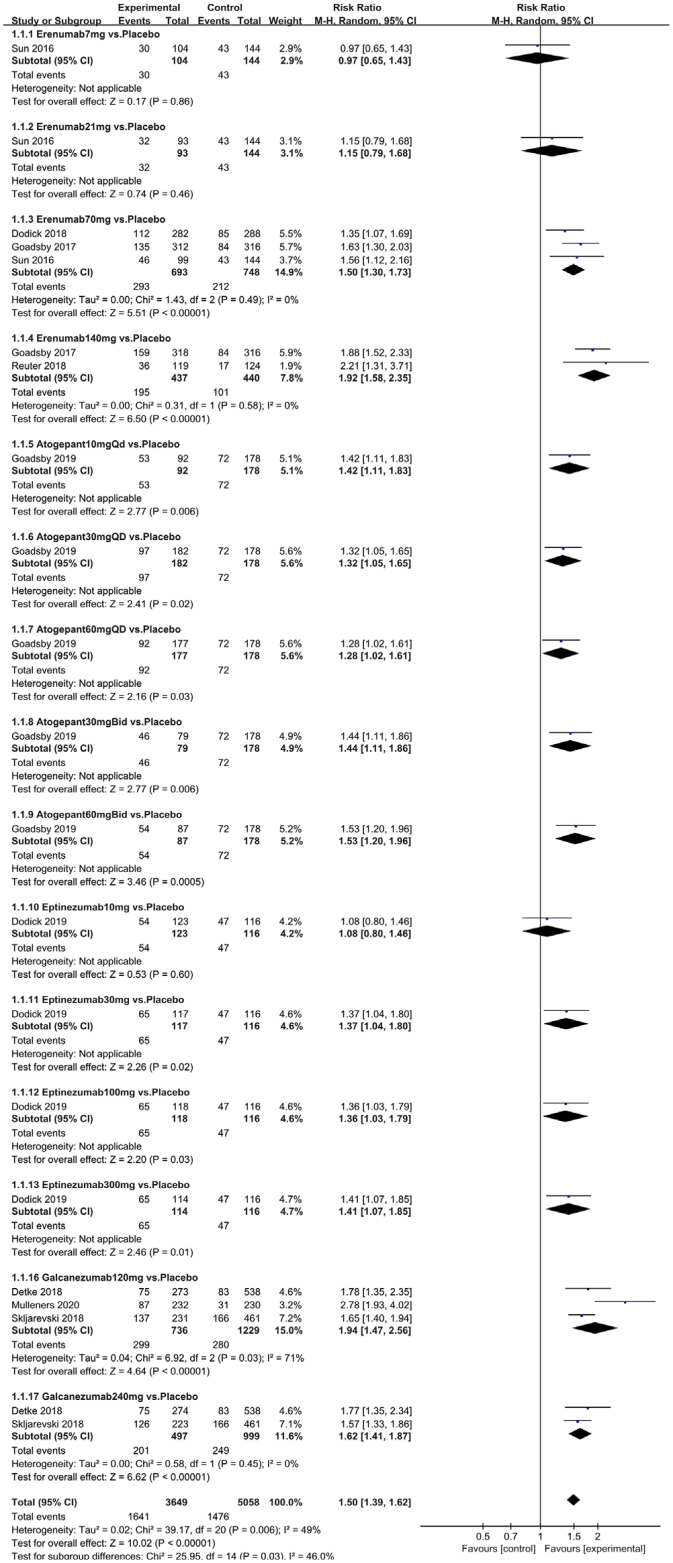
Forest of number of patients with ≥50% reduction from baseline in mean monthly migraine days in all groups

**FIGURE 3:**
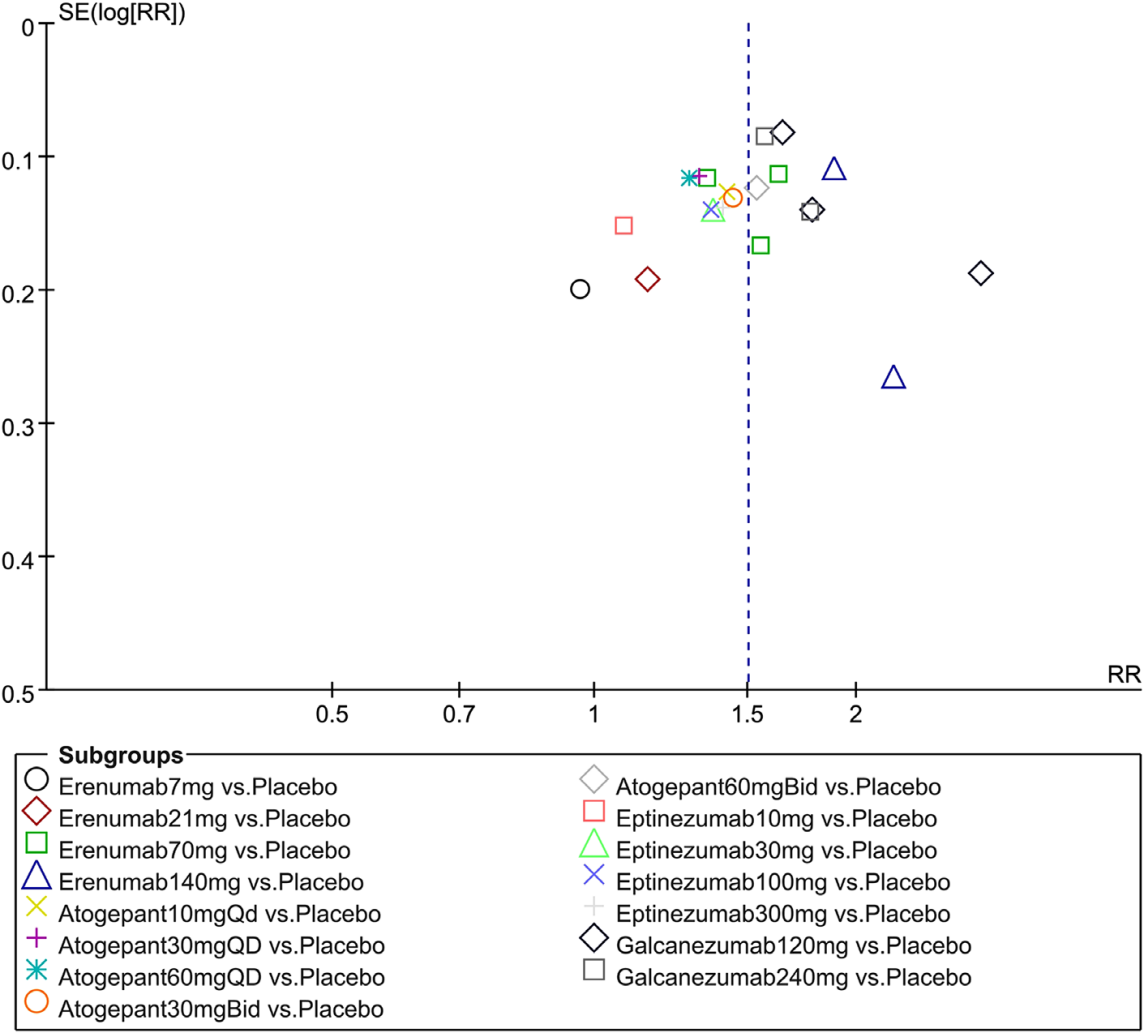
Funnel plot of number of patients with ≥50% reduction from baseline in mean monthly migraine days in all groups

**FIGURE 4:**
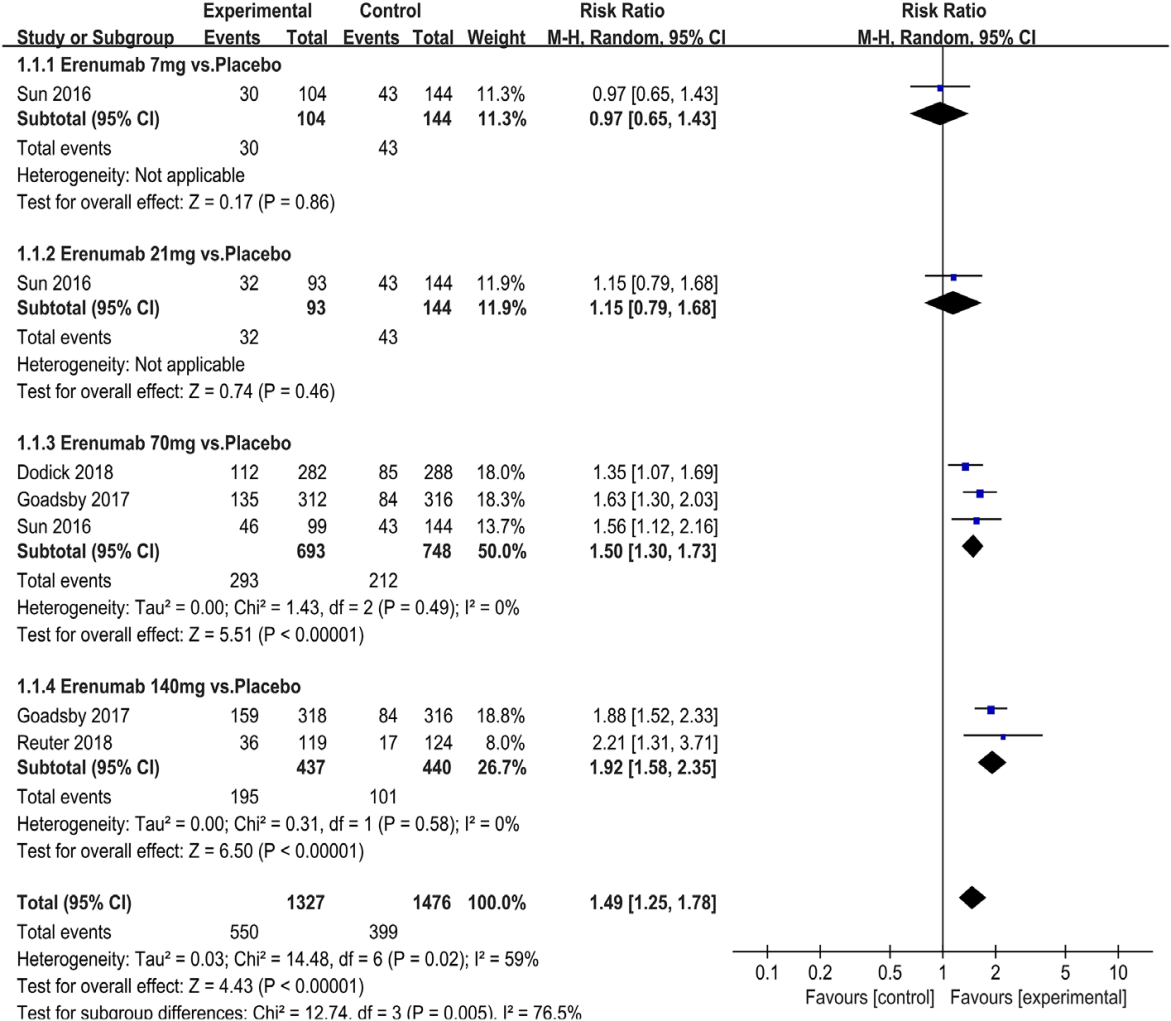
Forest of number of patients with ≥50% reduction from baseline in mean monthly migraine days in Erenumab group

**FIGURE 5:**
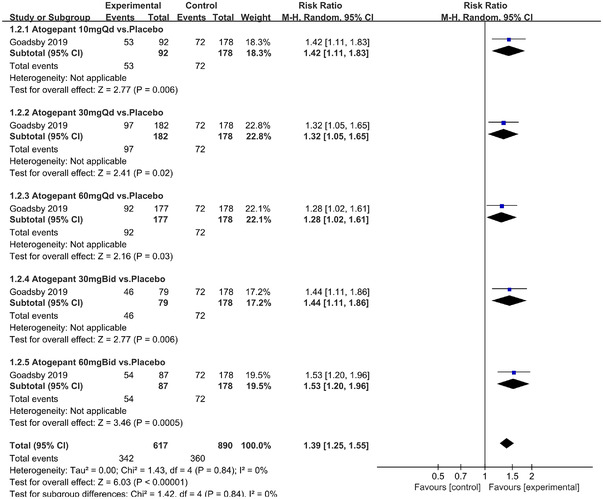
Forest of number of patients with ≥50% reduction from baseline in mean monthly migraine days in Atogepant group

**FIGURE 6:**
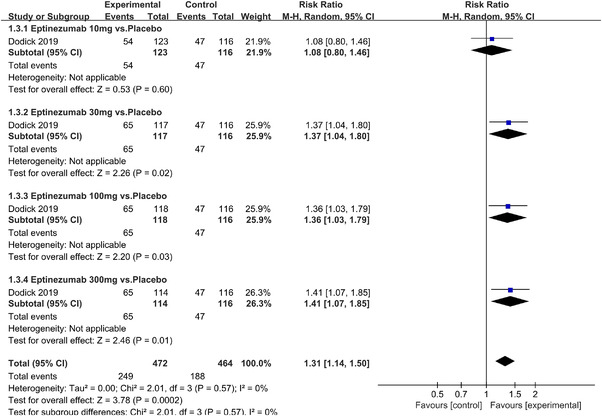
Forest of number of patients with ≥50% reduction from baseline in mean monthly migraine days in Eptinezumab group

**FIGURE 7:**
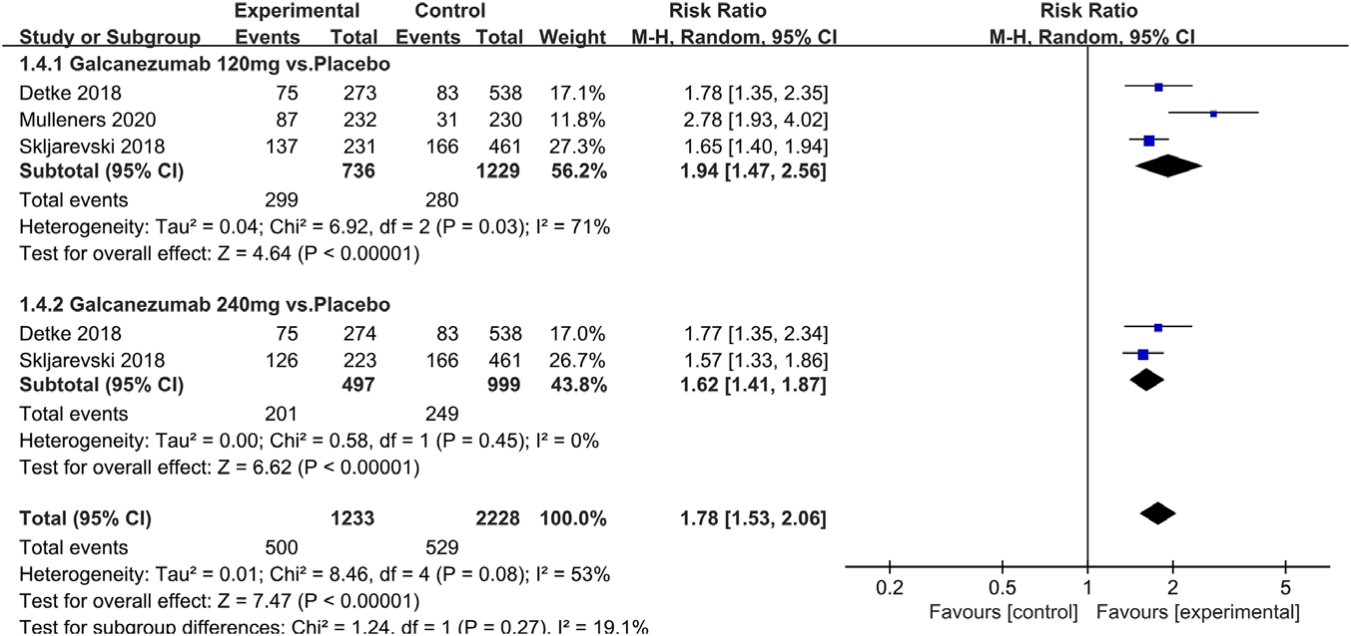
Forest of number of patients with ≥50% reduction from baseline in mean monthly migraine days in Galcanezumab group

**FIGURE 8:**
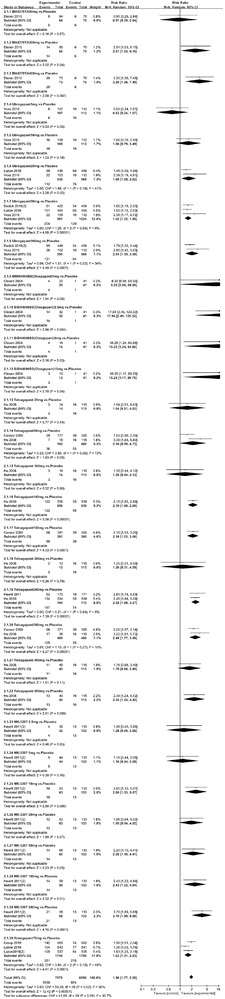
Forest of number of pain free patients at 2 h postdose in all groups

**FIGURE 9:**
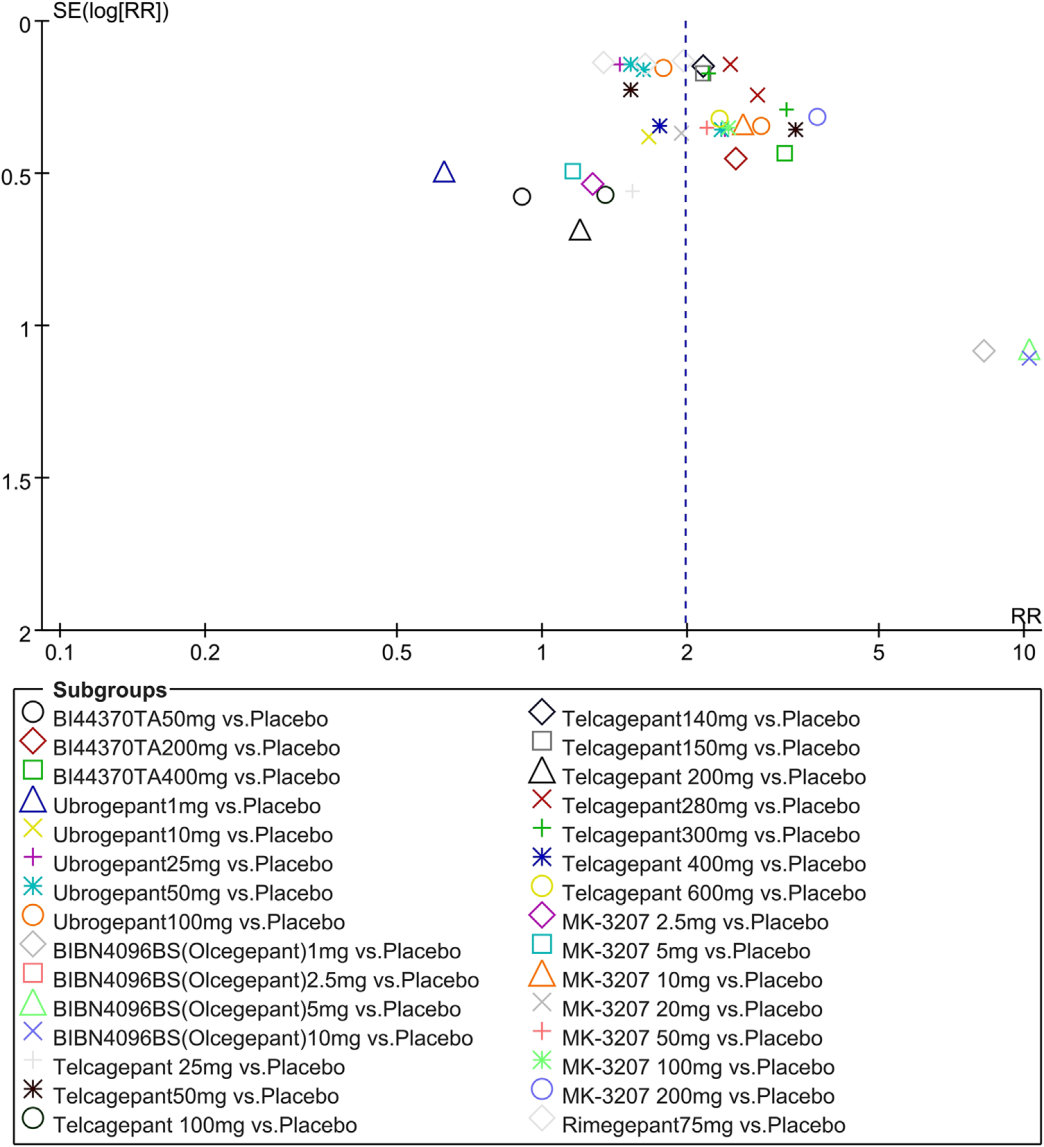
Funnel plot of number of pain free patients at 2 h postdose in all groups

**FIGURE 10:**
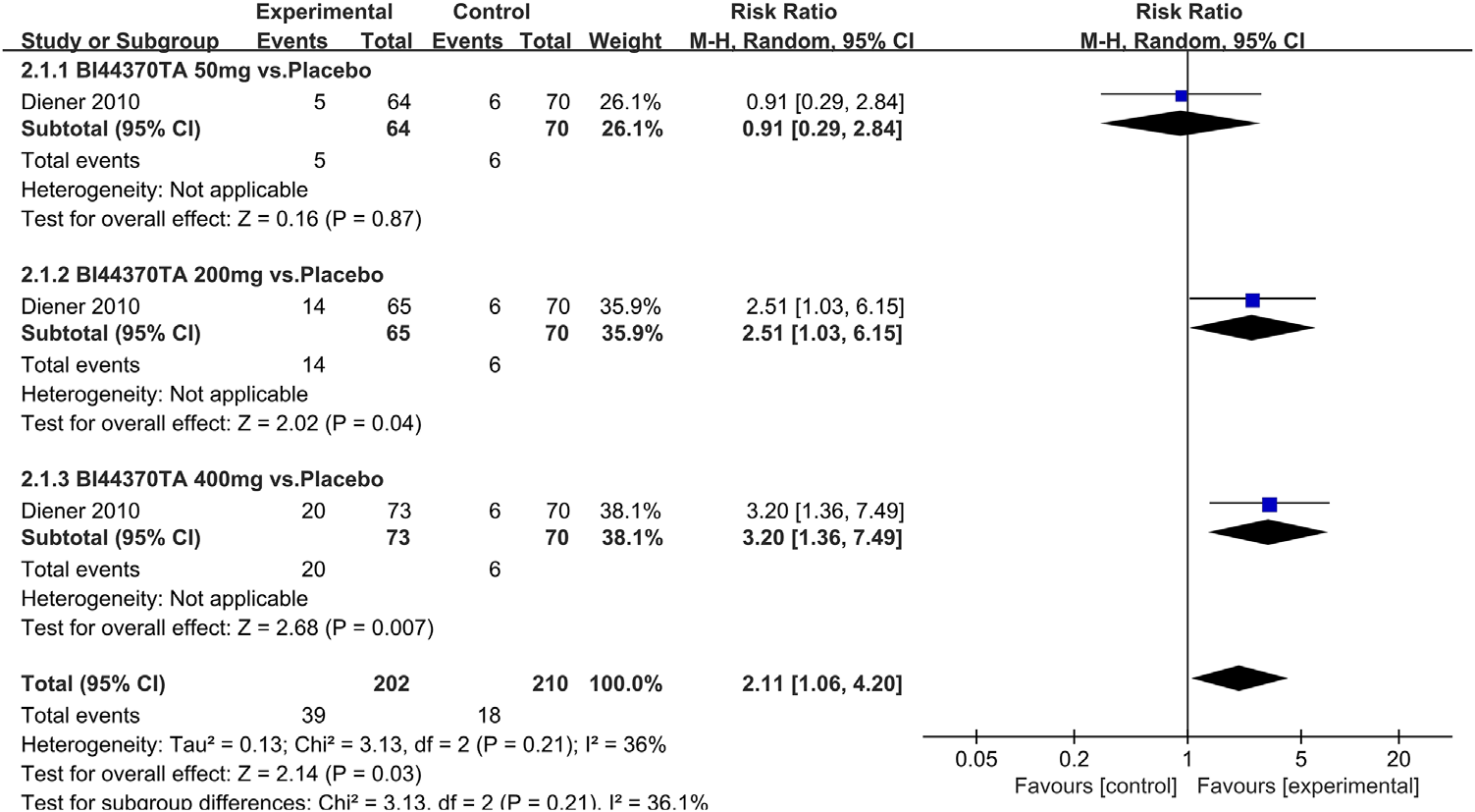
Forest of number of pain free patients at 2 h postdose in BI44370TA group

**FIGURE 11:**
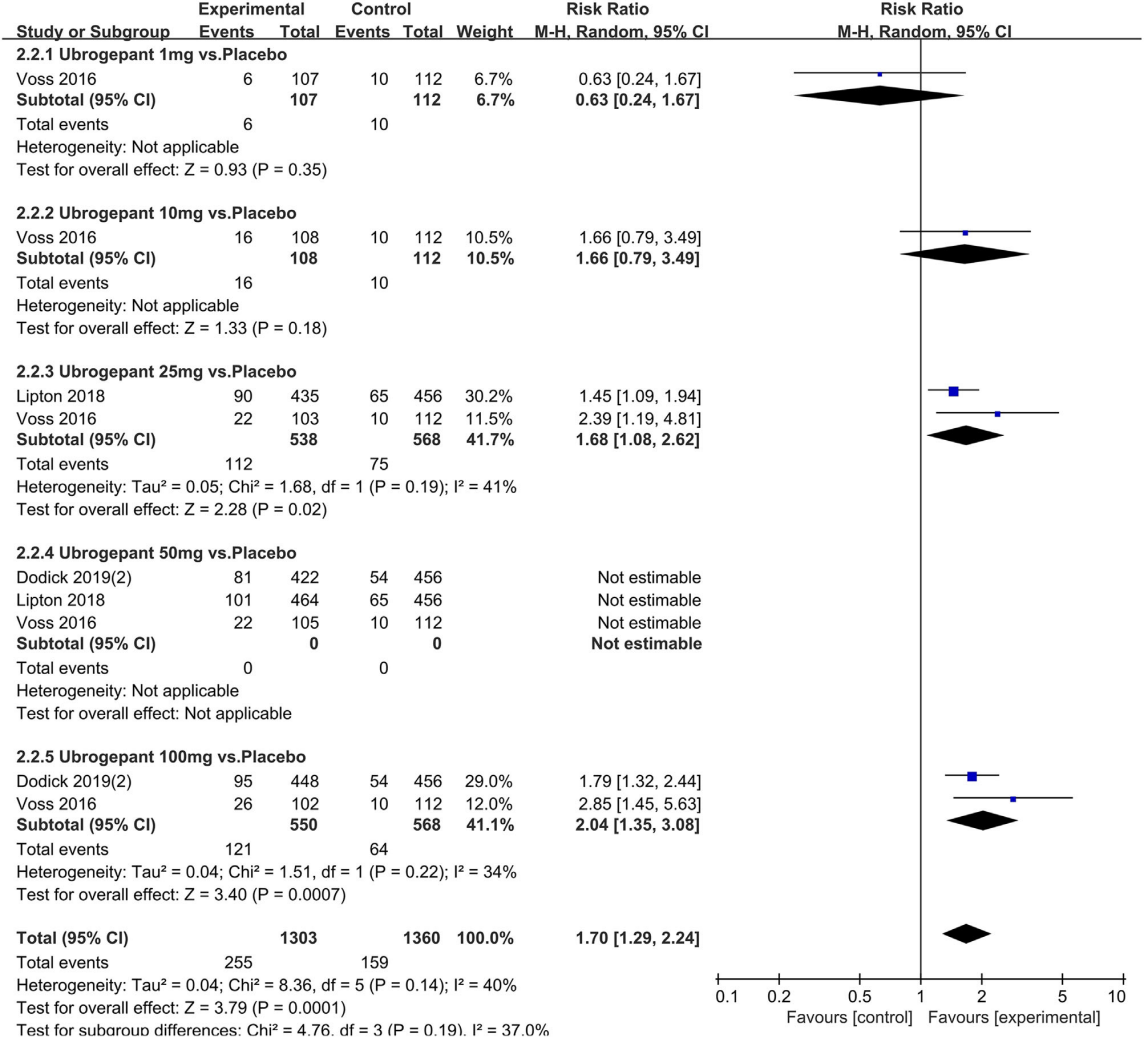
Forest of number of pain free patients at 2 h postdose in Ubrogepant group

**FIGURE 12:**
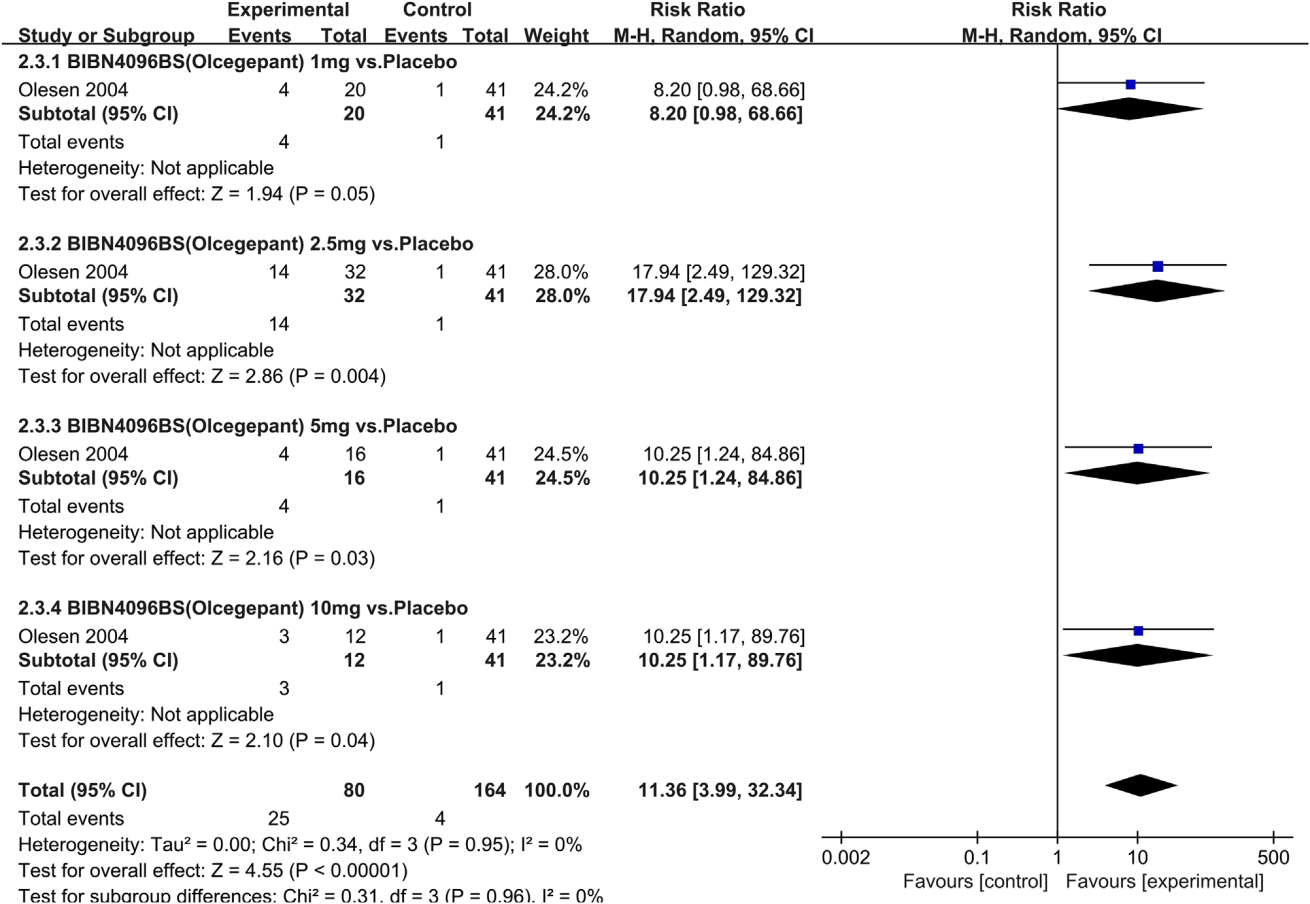
Forest of number of pain free patients at 2 h postdose in BIBN4096BS (Olcegepant) group

**FIGURE 13:**
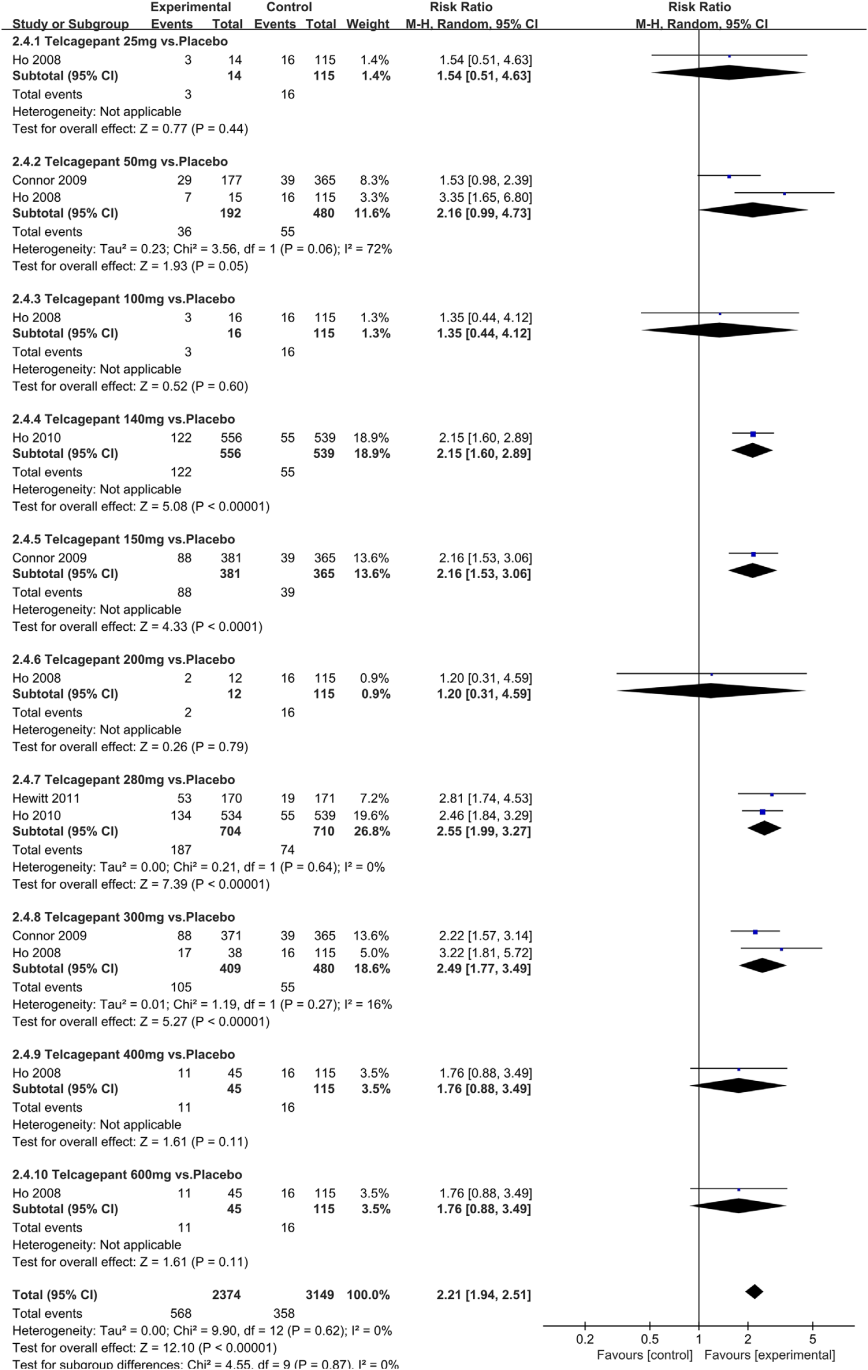
Forest of number of pain free patients at 2 h postdose in Telcagepant group

**FIGURE 14:**
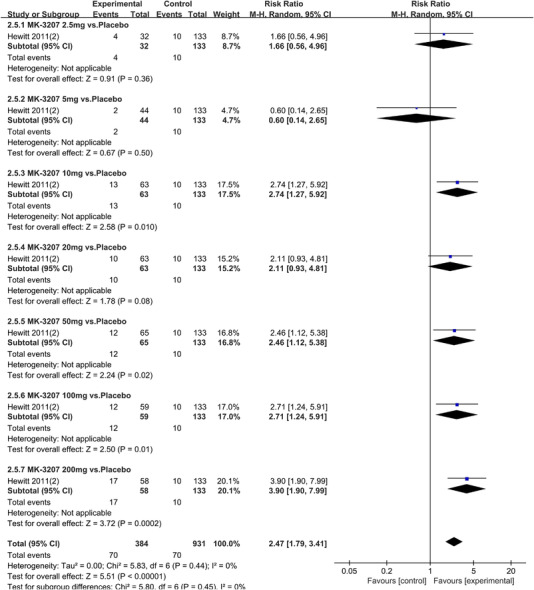
Forest of number of pain free patients at 2 h postdose in MK‐3207 group

**FIGURE 15:**
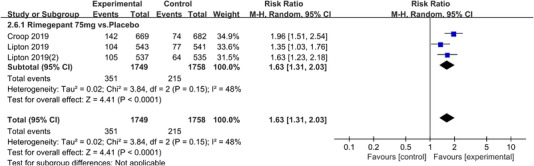
Forest of number of pain free patients at 2 h postdose in Rimegepant group

**FIGURE 16:**
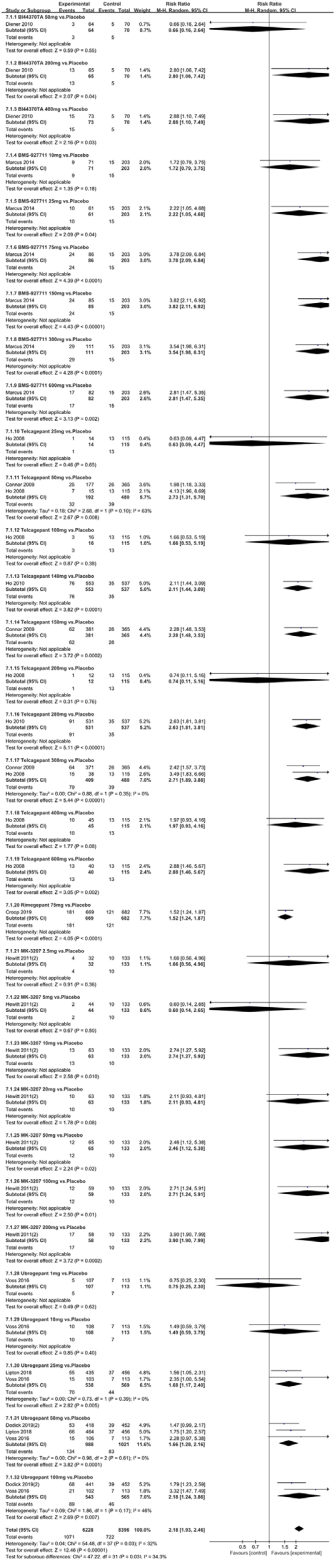
Forest of number of patients with sustained pain free 2–24 h postdose in all groups

**FIGURE 17:**
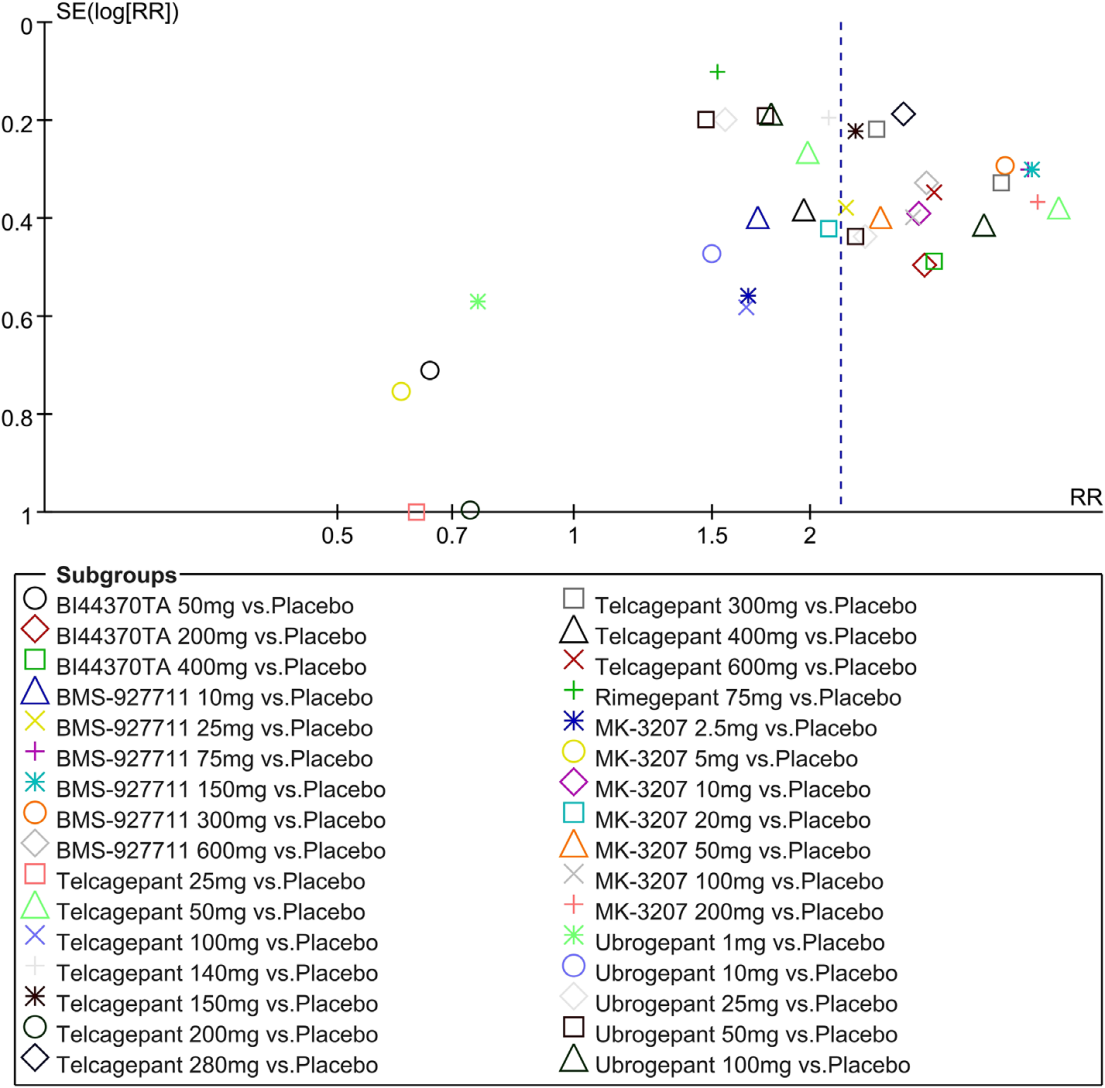
Funnel plot of number of patients with sustained pain free 2–24 h postdose in all groups

**FIGURE 18:**
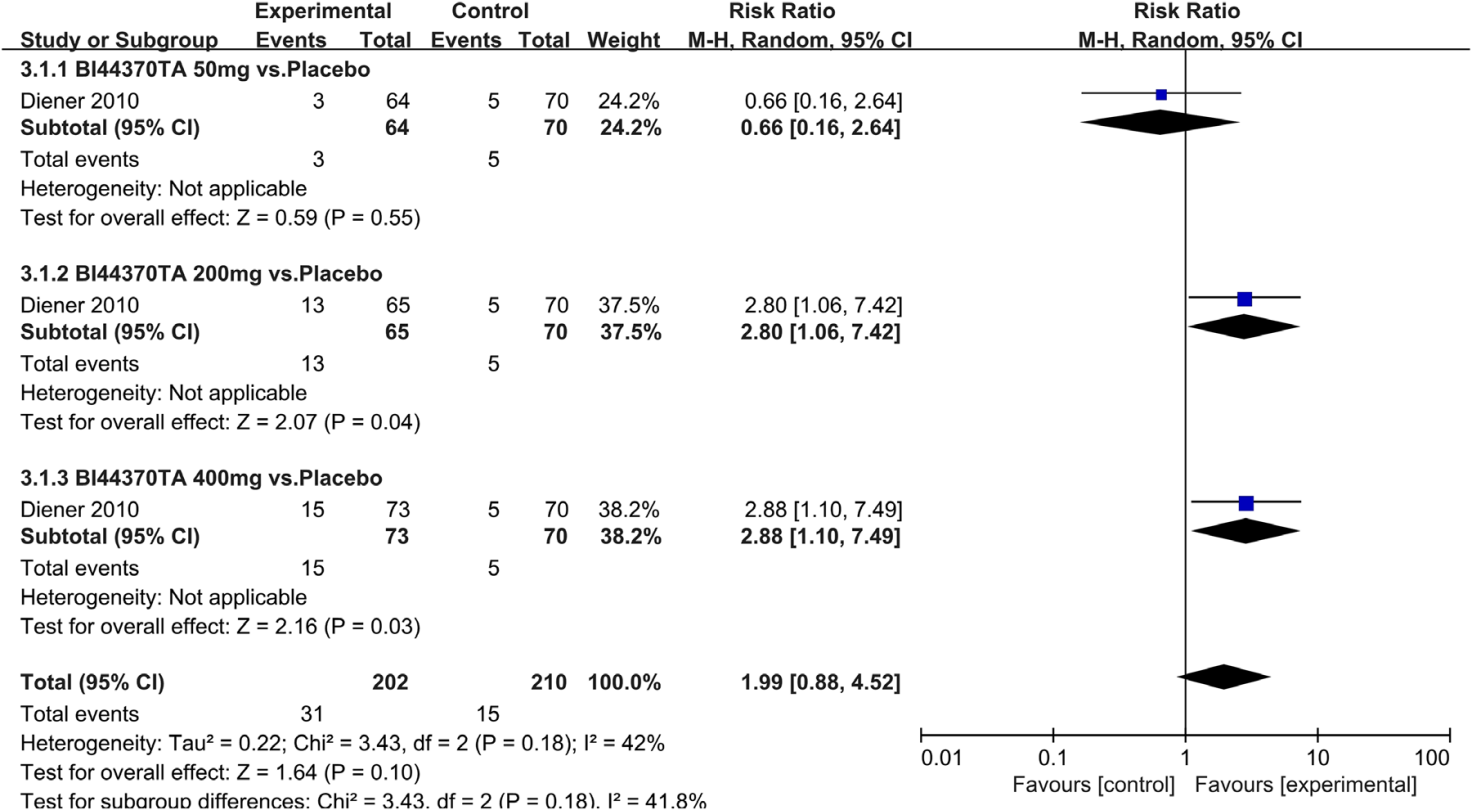
Forest of number of patients with sustained pain pain free 2–24 h postdose in BI44370TA group

**FIGURE 19:**
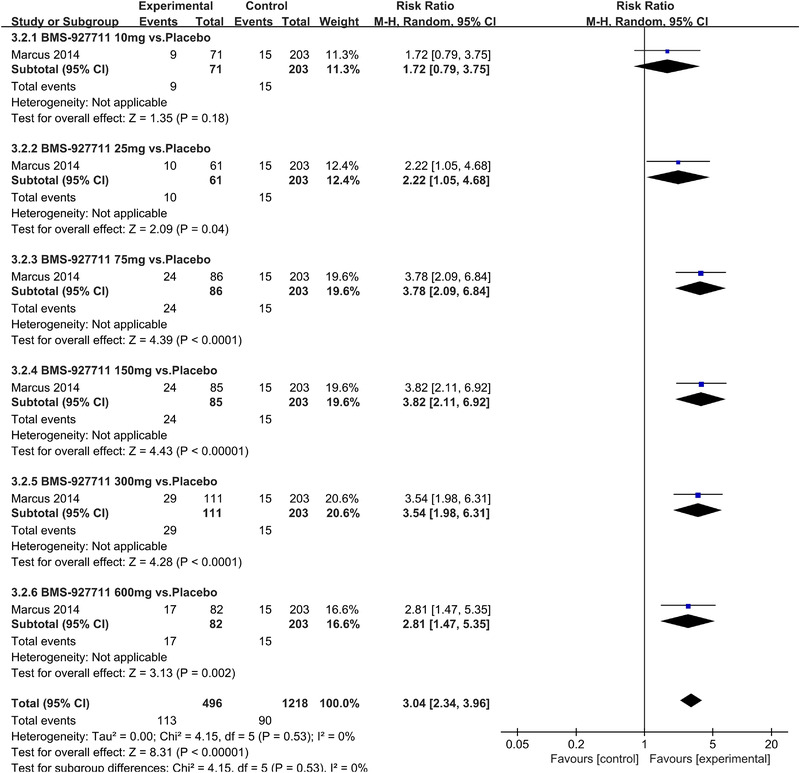
Forest of number of patients with sustained pain free 2–24 h postdose in BMS‐927711 group

**FIGURE 20:**
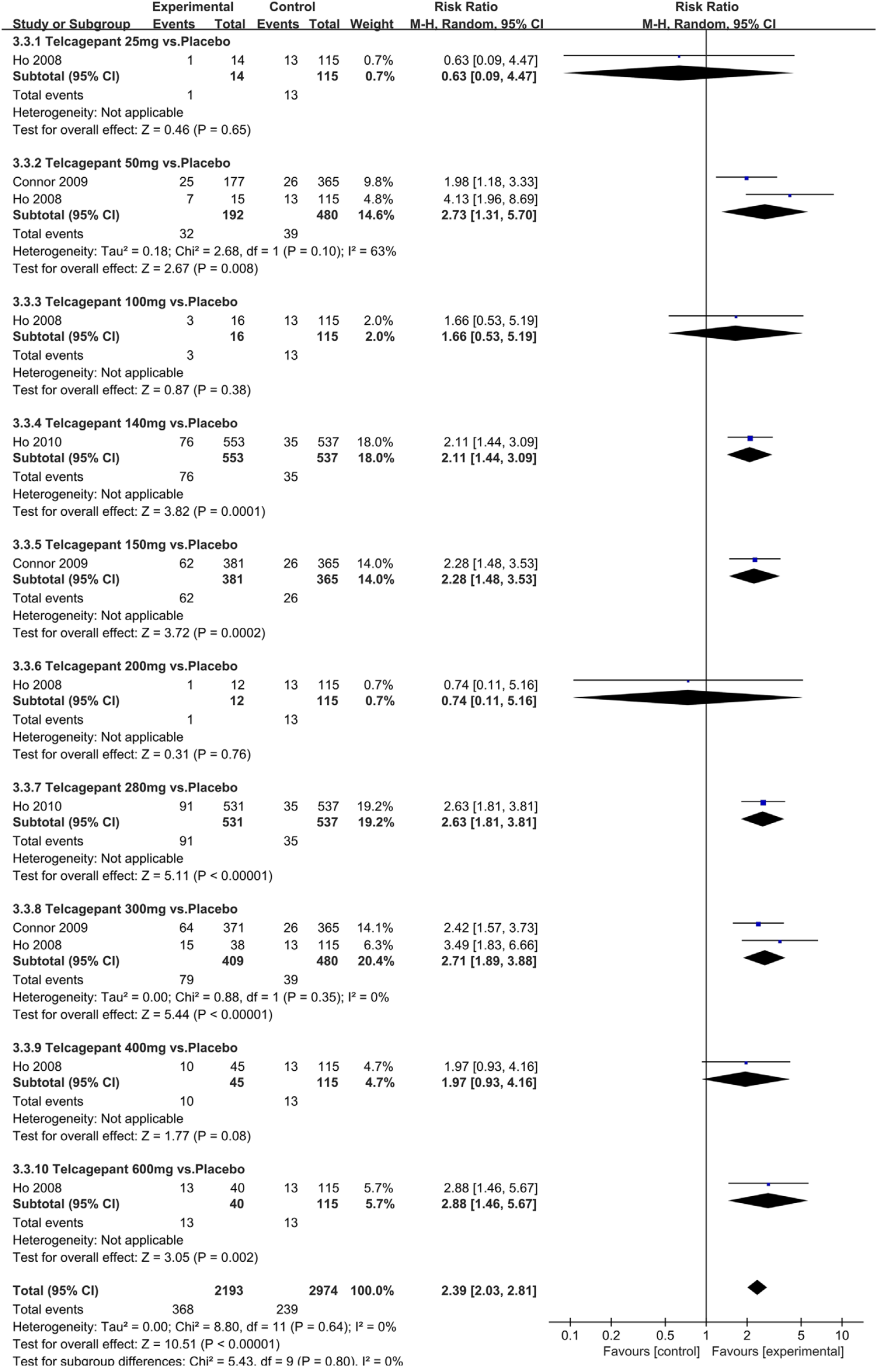
Forest of number of patients with sustained pain free 2–24 h postdose in Telcagepant group

**FIGURE 21:**
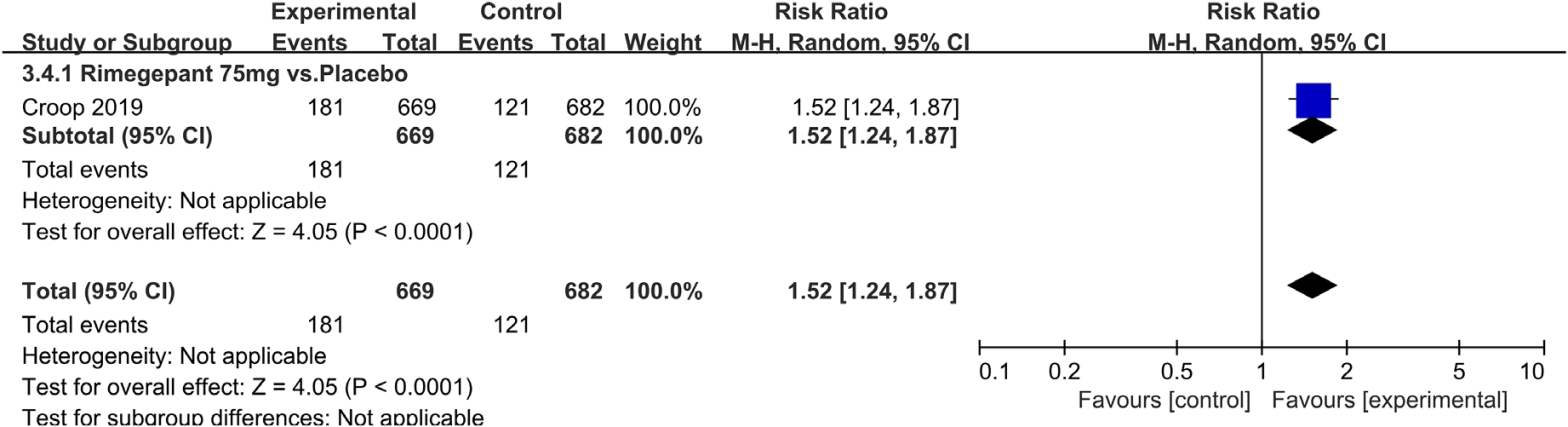
Forest of number of patients with sustained pain free 2–24 h postdose in Rimegepant group

**FIGURE 22:**
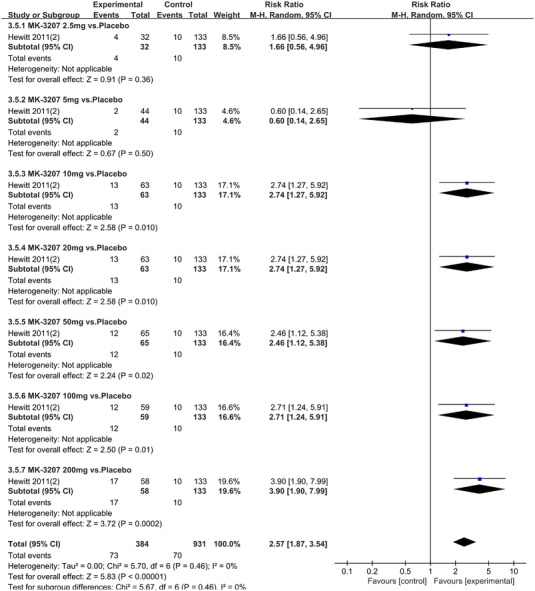
Forest of number of patients with sustained pain free 2–24 h postdose in MK‐3207 group

**FIGURE 23:**
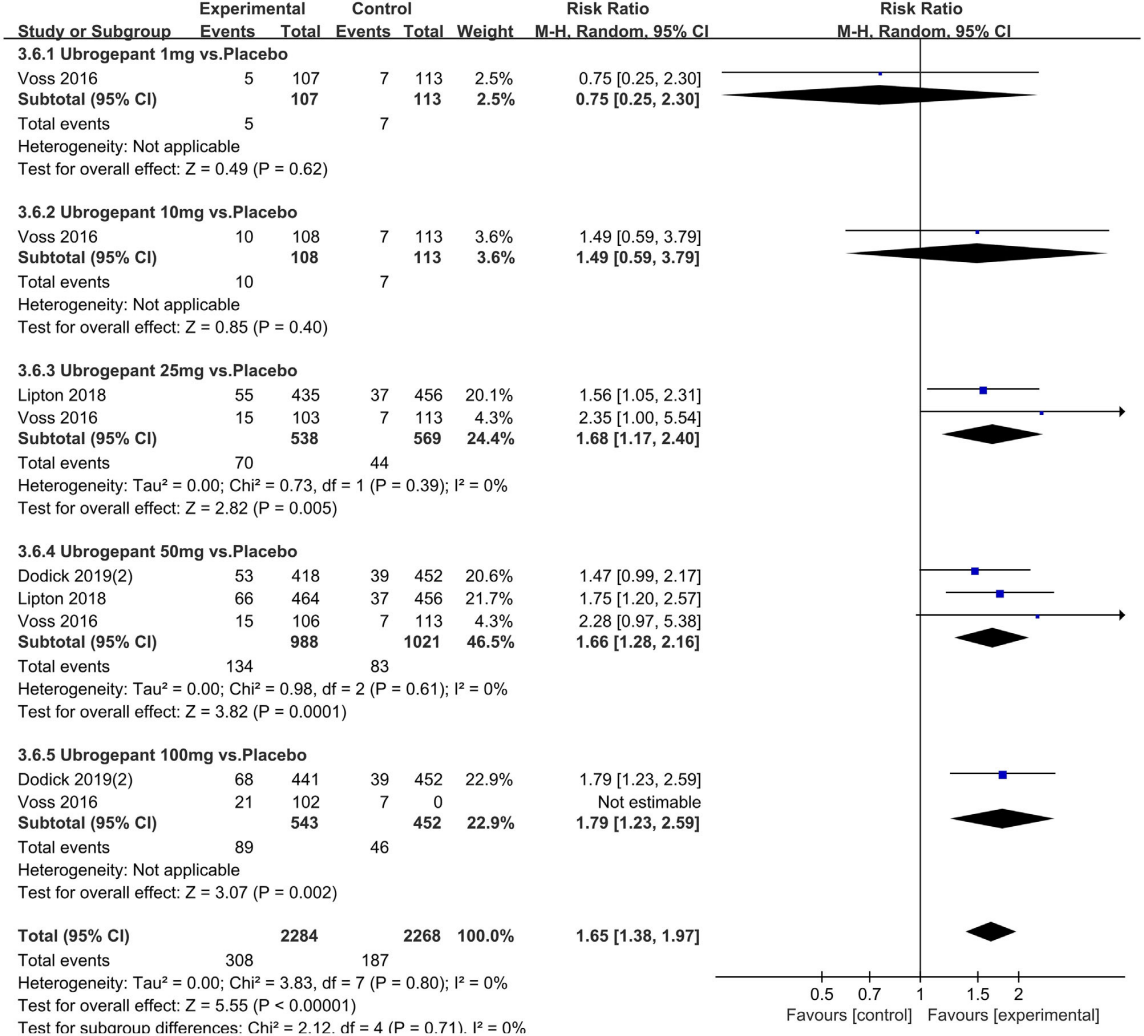
Forest of number of patients with sustained pain free 2–24 h postdose in Ubrogepant group

In terms of the three indicators, CGRP antagonists were found to be more effective when compared to controls. Outcome indicator for the number of patients with ≥50% reduction from baseline in mean monthly migraine days was reported in 9 RCTs and the random effects model showed that CGRP antagonists exerted better effects, relative to controls (RR = 1.50, 95% CI [1.39, 1.62], *p* < .00001) (Figures 2 and 3) and the meta‐analysis of each CGRP antagonist for the outcome indicator of “the number of patients with ≥50% reduction from baseline in mean monthly migraine days” were also performed (Figures 4, 5, 6, 7). Outcome indicator for the number of pain free patients at 2 h postdose was reported in 13 RCTs and the random effects model showed that CGRP antagonists exerted better effects relative to the controls (RR = 1.98, 95% CI [1.77, 2.20], *p* < .00001) (Figures 8 and 9) and the meta‐analysis of each CGRP antagonist for the outcome indicator of “number of pain free patients at 2 h postdose” were also performed (Figures 10, 11, 12, 13, 14, 15). Outcome indicator for the number of patients with pain‐free patients at 2–24 h postdose was reported in 10 RCTs and the random effects model showed that CGRP antagonists exerted better effects, relative to controls (RR = 2.18, 95% CI [1.93, 2.46], *p* < .00001) (Figures 16 and 17) and the meta‐analysis of each CGRP antagonist for the outcome indicator of “number of patients with pain‐free patients at 2–24 h postdose” were also performed (Figures 18, 19, 20, 21, 22, 23).

#### 3.3.2 Safety outcomes

**FIGURE 24:**
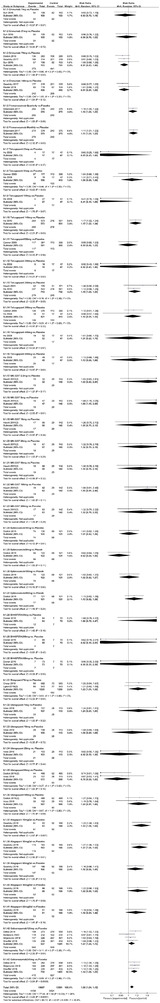
Forest of AEs in all groups

**FIGURE 25:**
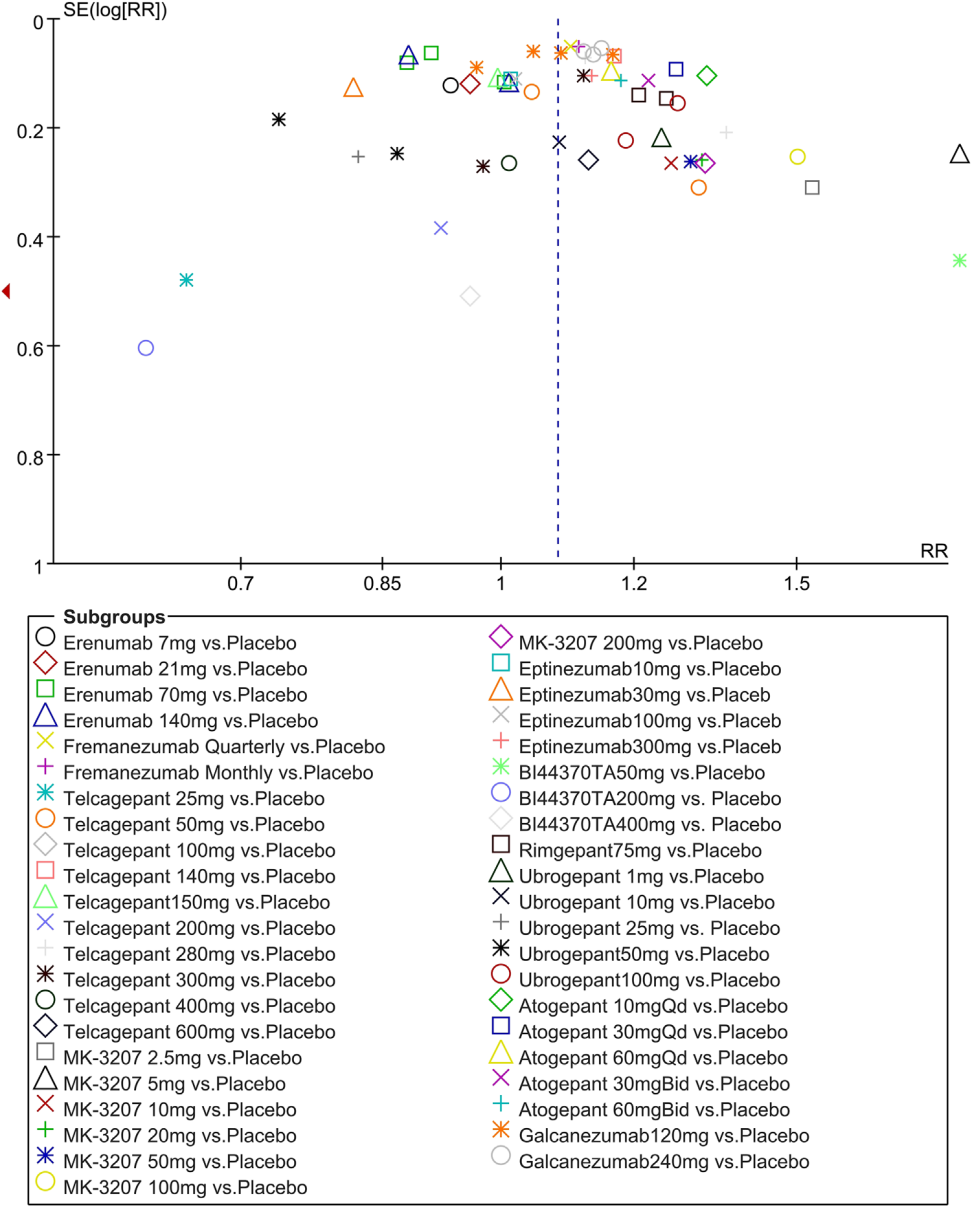
Funnel plot of AEs in all groups

**FIGURE 26:**
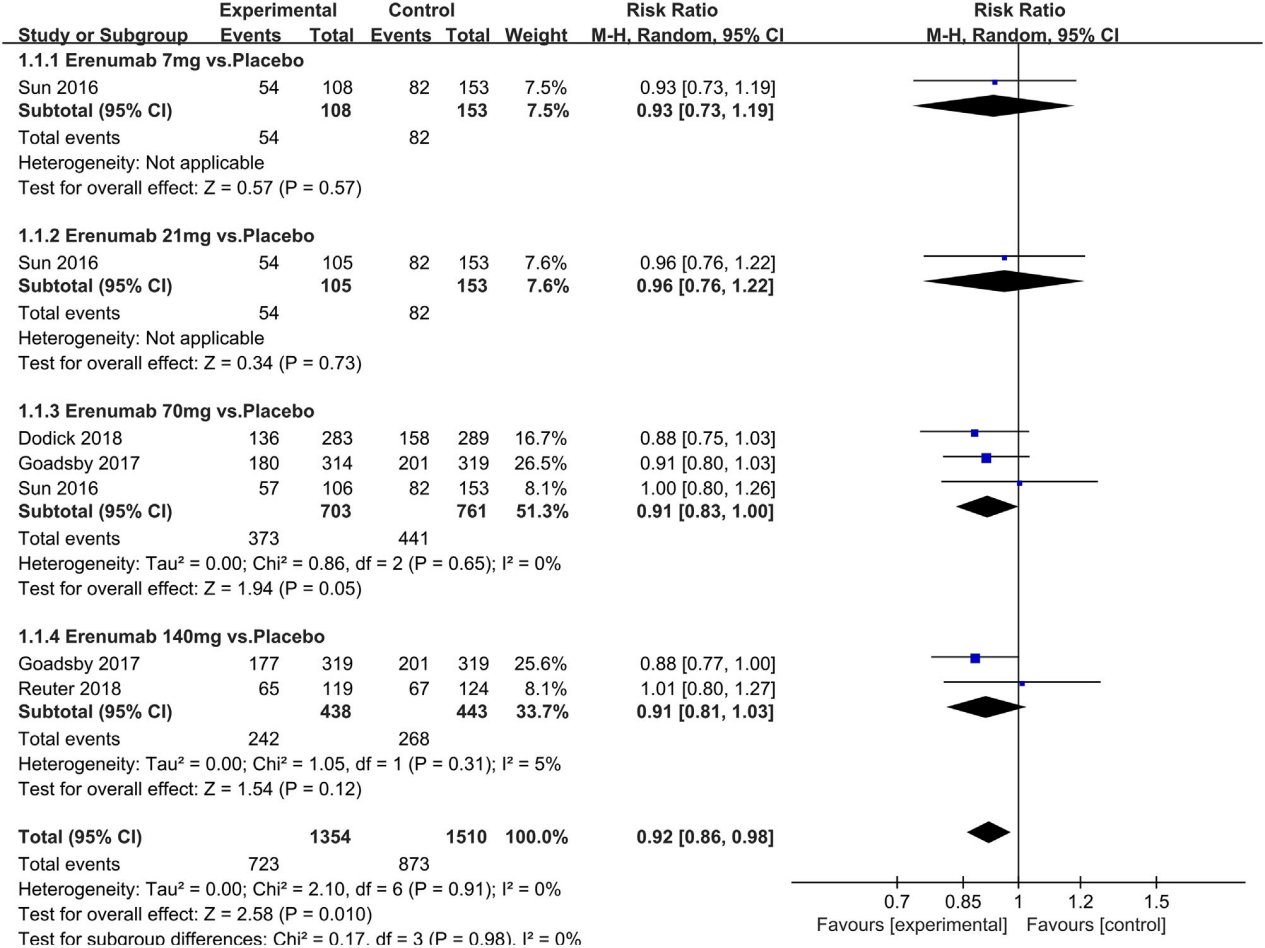
Forest of AEs in Erenumab group

**FIGURE 27:**
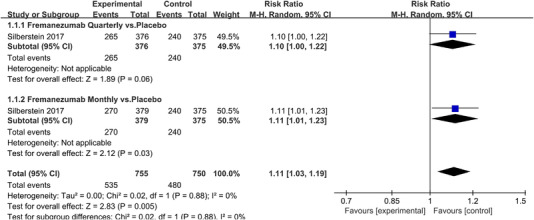
Forest of AEs in Fremanezumab group

**FIGURE 28:**
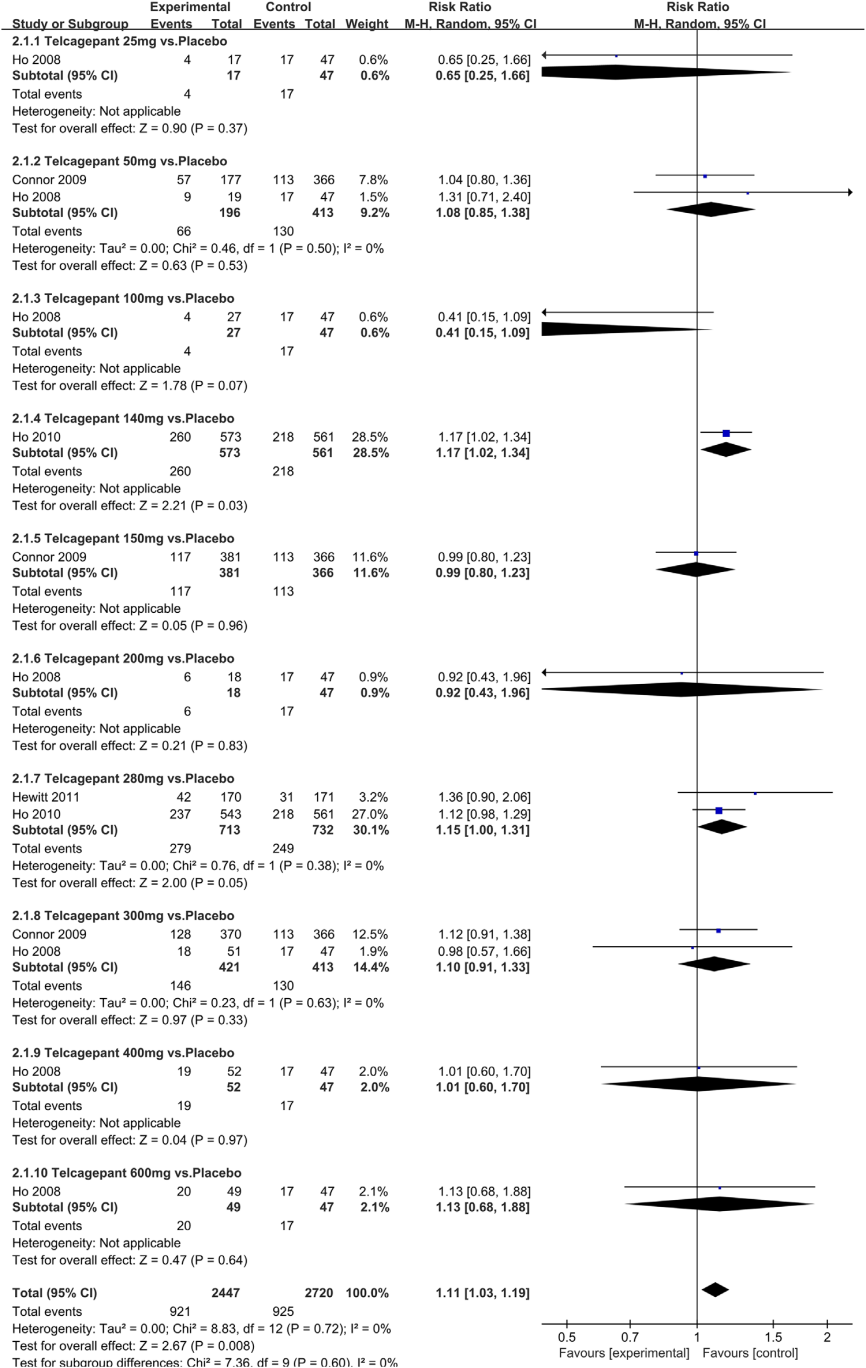
Forest of AEs in Telcagepant group

**FIGURE 29:**
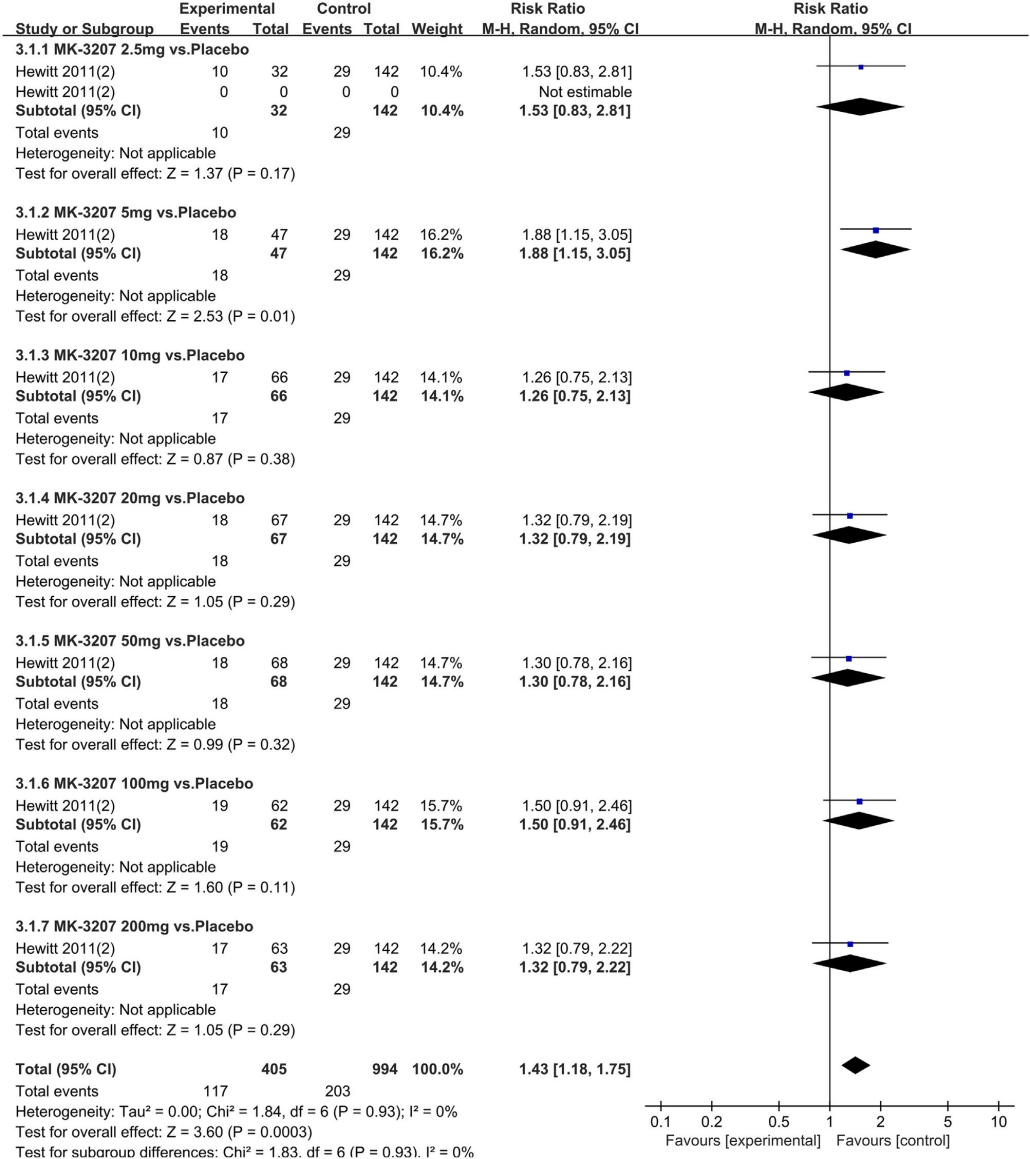
Forest of AEs in MK‐3207 group

**FIGURE 30:**
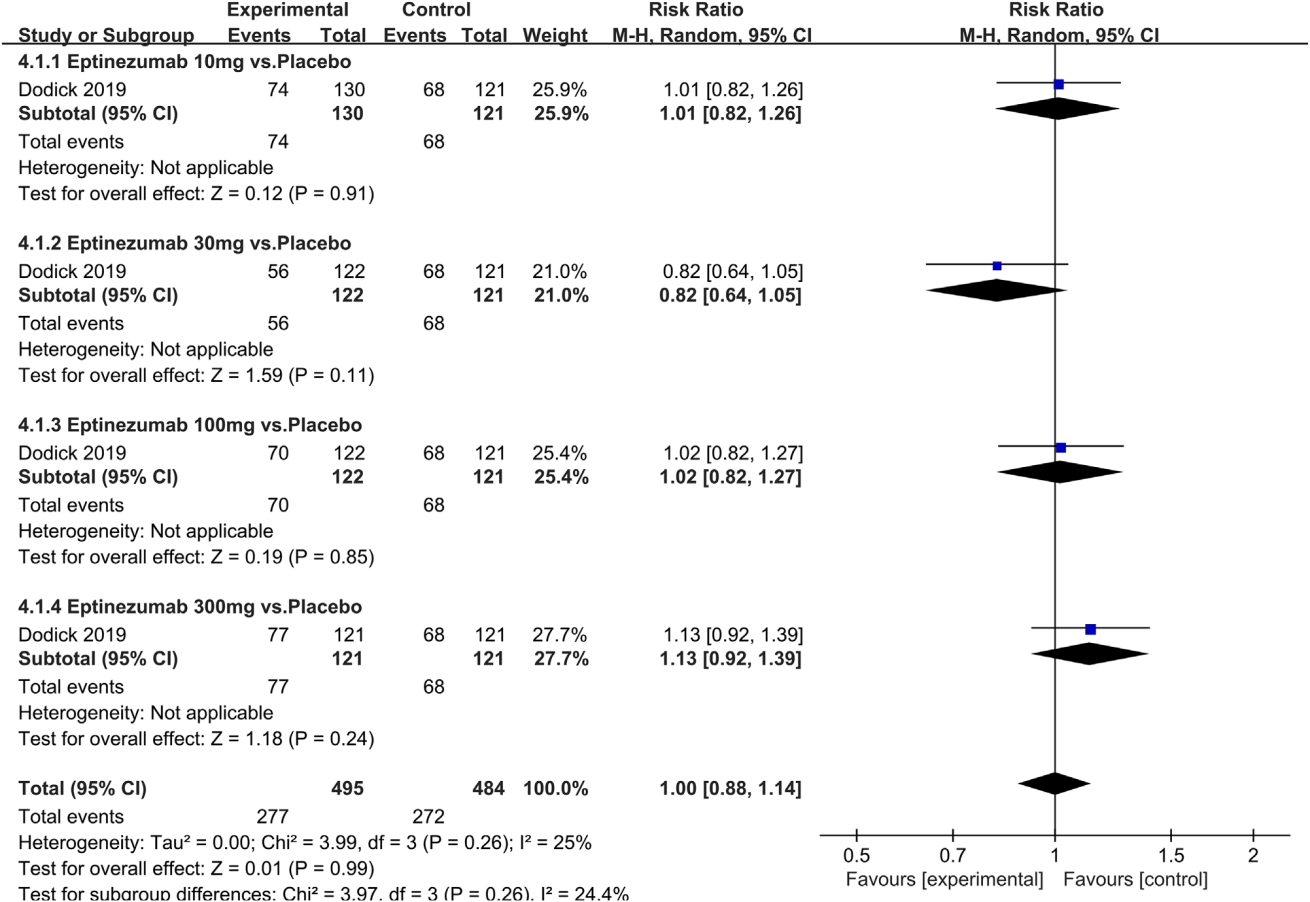
Forest of AEs in Eptinezumab group

**FIGURE 31:**
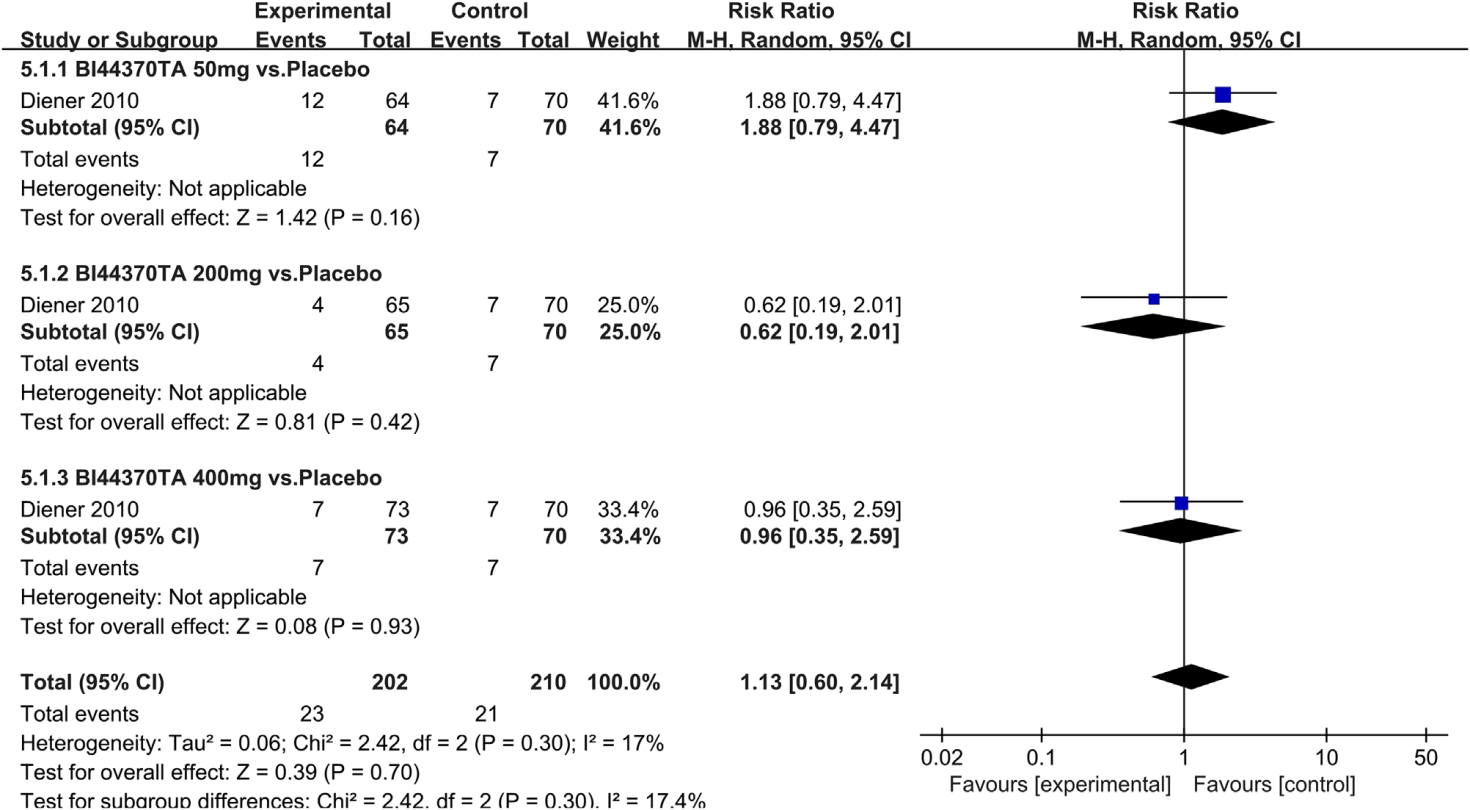
Forest of AEs in Bl44370TA group

**FIGURE 32:**
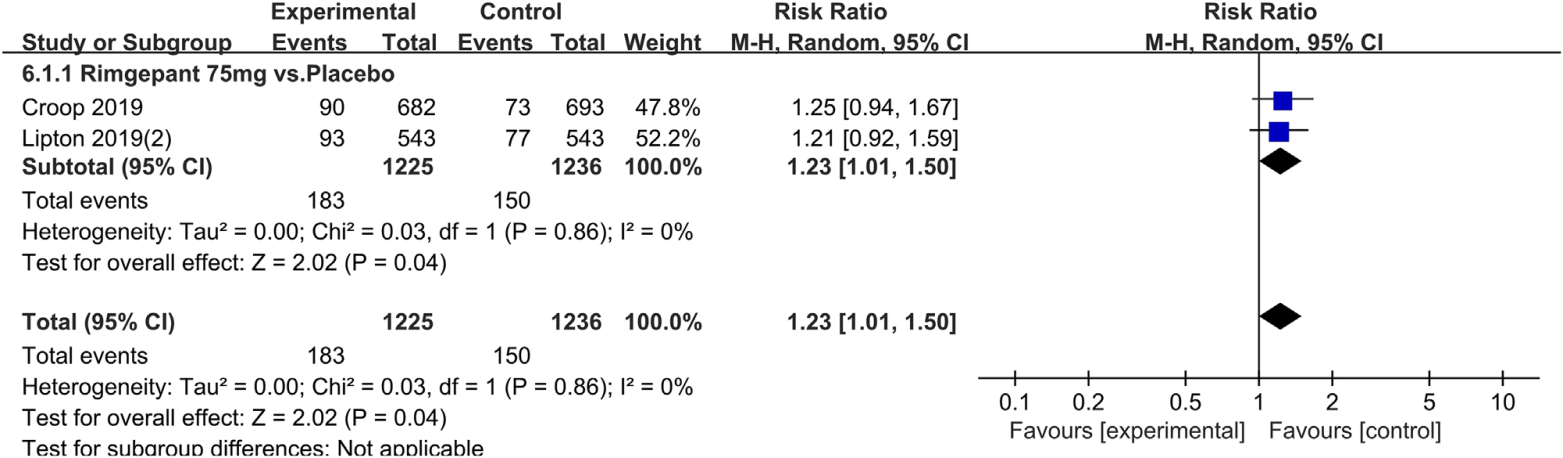
Forest of AEs in Rimgepant group

**FIGURE 33:**
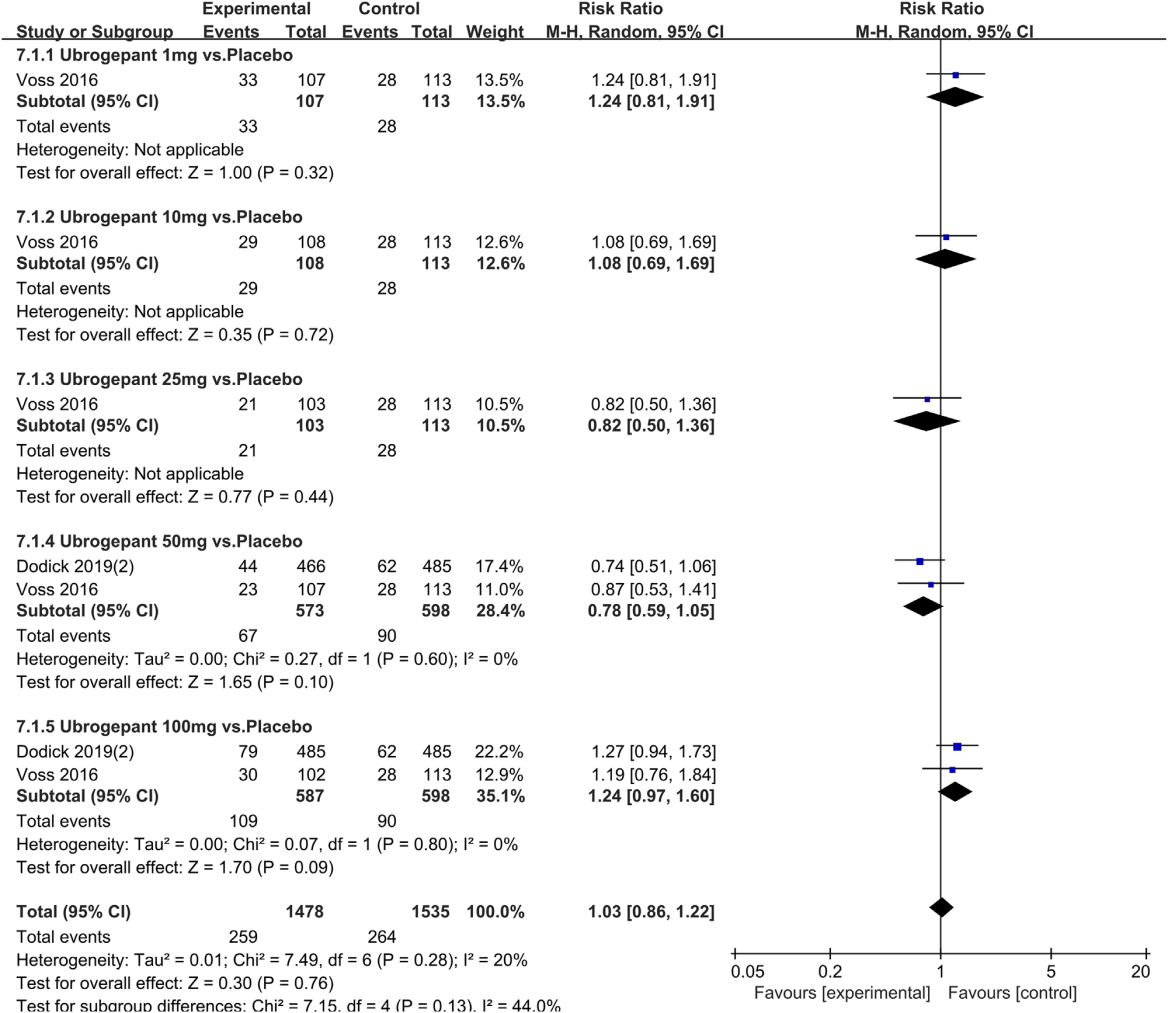
Forest of AEs in Ubrogepant group

**FIGURE 34:**
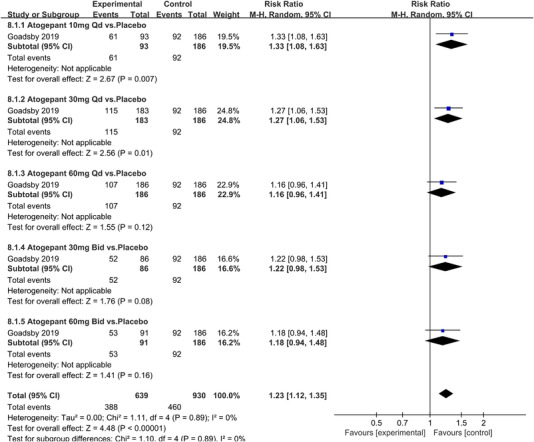
Forest of AEs in Atogepant group

**FIGURE 35:**
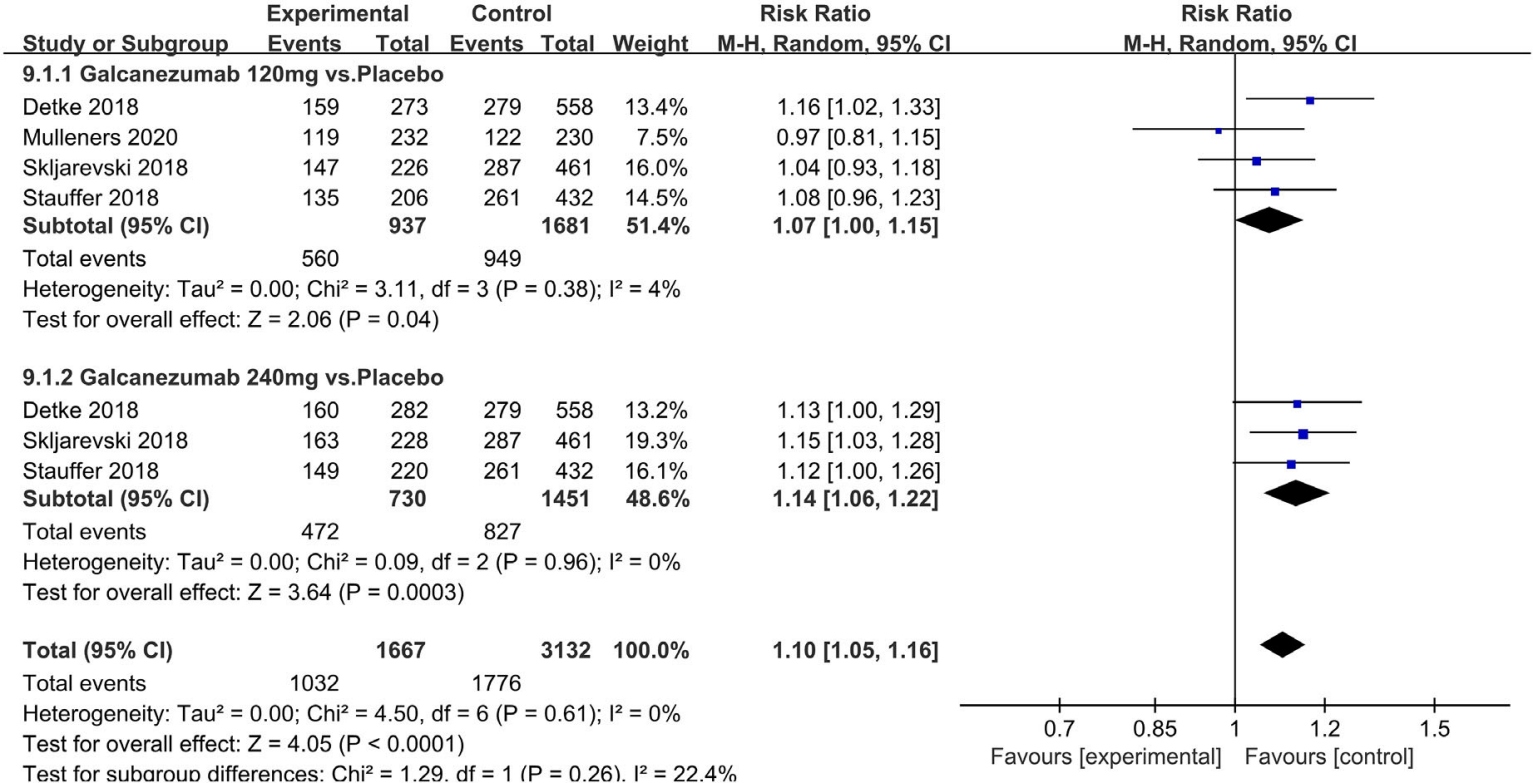
Forest of AEs in Galcanezumab group

Twenty‐one RCTs reported on treatment‐associated adverse outcomes. The random effects model showed that CGRP antagonists were associated with more adverse reactions than controls (RR = 1.08, 95% CI [1.04, 1.12], *p* < .0001) (Figures 24 and 25) and the meta‐analysis of each CGRP antagonist for the adverse outcomes were also performed (Figures 26, 27, 28, 29, 30, 31, 32, 33, 34, 35). The adverse reactions included upper respiratory tract infections, pain at injection sites (dose dependent), nasopharyngitis, influenza, fatigue, somnolence, nausea, and constipation among others.

#### 3.3.3 Sensitivity analysis

**FIGURE 36:**
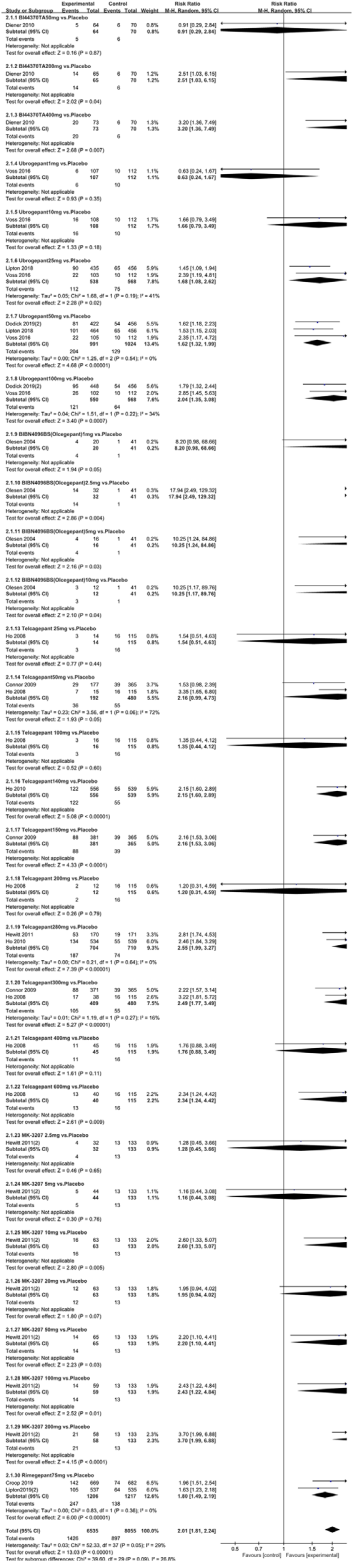
Forest of number of pain free patients at 2 h postdose in all groups excluding one RCT (Lipton, Croop et al., 2019)

**FIGURE 37:**
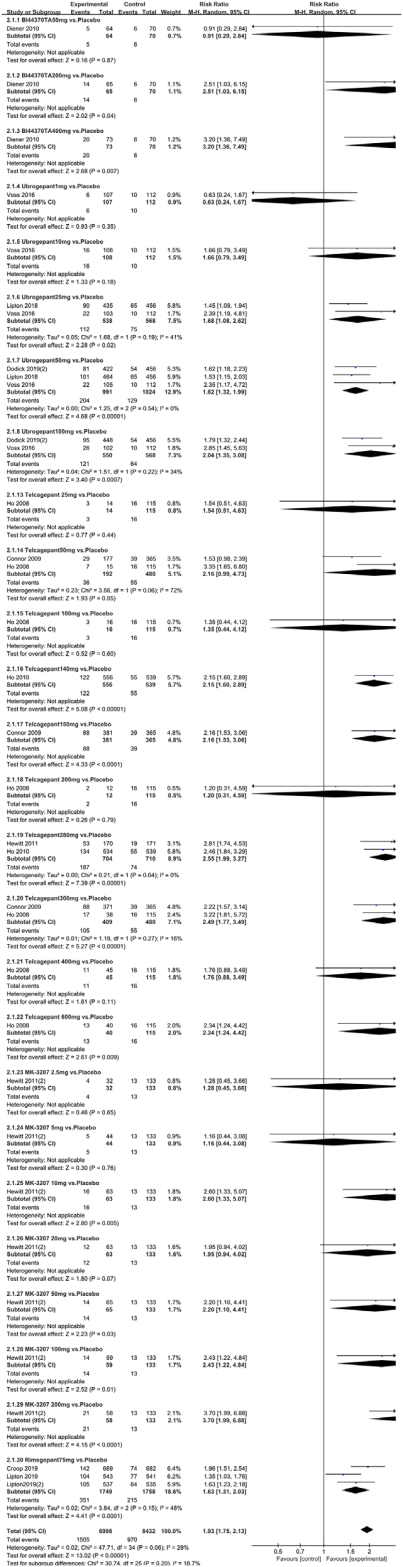
Forest of number of pain free patients at 2 h postdose in all groups excluding one RCT (Olesen et al., 2004)

To check the stability of the results obtained from this meta‐analysis, leave‐one out method was used by analyzing the results when deleting one single trial each time. We first excluded the lowest quality RCT (Lipton, Croop et al., 2019), which got “2” scores by Jadad Scale and then performed the meta‐analysis again. It showed that the outcome indicator of “number of pain free patients at 2 h postdose” was still in line with the original result (RR = 2.01, 95% CI [1.81, 2.24], *p* < .00001, Figure 36). Then we excluded the RCT (Olesen et al., 2004) for the reason of small sample size. And we also found the consistency with the original result on the outcome indicator of “number of pain free patients at 2 h postdose” (RR = 1.93, 95% CI [1.75, 2.13], *p* < .00001, Figure 37).

## 4 DISCUSSION

The trigeminal neurovascular theory states that the trigeminal ganglion is a trigeminal nerve vascular reflex center that once activated, it can cause pain perception in migraine patients. CGRP is expressed in C fibers of the trigeminal ganglion, while CGRP receptors are expressed in the Aδ fiber. These fibers play different roles in pain induction. Since the trigeminal ganglion does not have a blood‐brain barrier with endocranium, it is a potential drug target. CGRP antagonists have been shown to relieve pain in migraine patients (Edvinsson et al., 2018). This meta‐analysis is presented based on the types and doses of CGRP antagonists. We found that CGRP antagonists exerted better effects relative to the controlled group in which conventional drugs, such as NSAIDS, triptans, or placebo were administered to migraine patients. These outcomes indicators included (1) number of patients with ≥50% reduction from baseline in mean monthly migraine days (RR = 1.50, 95% CI [1.39, 1.62], *p* < .00001);(2) number of pain free patients at 2 h post‐dosing (RR = 1.98, 95% CI [1.77, 2.20], *p* < .00001); (3) number of sustained pain‐free patients at 2–24 h sustained post‐dosing (RR = 2.18, 95% CI [1.93, 2.46], *p* < .00001). And though subgroup analysis, all CGRP antagonist groups showed better outcomes than controls. BI44370TA, ubrogepant, telcagepant, MK‐3207, and rimegepant showed better outcomes both on outcomes including number of patients with pain free at 2 h postdose and number of patients with 2–24 h sustained pain free postdose. However, CGRP antagonists were associated with more adverse reactions than controls (RR = 1.08, 95% CI [1.04, 1.12], *p* < .0001), which was not consistent with previous studies; the reasons may be that (1) previous meta‐analyses that evaluated the efficacy and safety of calcitonin gene‐related peptide antagonists for migraine treatment focused on only one type of CGRP antagonists, such as small molecule CGRP receptor antagonists or anti‐CGRP monoclonal antibodies, while this meta‐analysis focused on both small molecule CGRP receptor antagonists and anti‐CGRP monoclonal antibodies. (2) Anti‐CGRP monoclonal antibodies are mostly administered via injections, which can easily lead to injection related adverse reactions, such as localized redness and swelling of the skin. (3) RCTs on CGRP antagonists always classified drugs based on dose gradients, which can lead to dose‐dependent adverse reactions, such as somnolence, nausea and vomiting among others. However, the above adverse reactions are mild and transient and do not cause much harm to patients’ health. Only one death was reported in the fremanezumab quarterly group set by silberstein and the cause of death was determined to be chronic obstructive pulmonary disease (COPD) (Silberstein et al., 2017). Compared to small molecule CGRP receptor antagonists (gepants), anti‐CGRP monoclonal antibodies (mAbs) are not associated with hepatotoxic effects because they are not metabolized in the liver. Moreover, mAbs have long half‐lives, high affinity as well as selectivity and they do not exert adverse effects on the cardiovascular system, which is a common adverse reaction site for triptan. By the way, through meta‐analysis of each drug, erenumab showed less adverse reaction than control and eptinezumab showed much the same adverse reaction with control, and these two CGRP antagonists also showed greater effect on outcome of “number of patients with ≥50% reduction from baseline in mean monthly migraine days” compared with controls, which can provide guidance to clinical work.

## 5 LIMITATIONS

This meta‐analysis has some limitations: (1) the included participants in most studies were almost middle‐aged and young, with a majority of them being women and (2) the maximum follow‐up time was 256 weeks, which is comparatively short to explore the long‐term efficacies and safety of the CGRP antagonists on migraine treatment. Based on the above limitations, future studies should evaluate the efficacies of CGRP antagonists among different age groups combined with other diseases, the benefits and safety of long‐term drug use, withdrawal reactions on the vascular system, and the stability of drug responses with multiple migraine attacks.

## 6 CONCLUSION

This meta‐analysis reveals that CGRP antagonists can be effective drug for migraine treatment. However, their safety need more high‐quality RCT researches to be proved.

## AUTHOR CONTRIBUTIONS

Tingting Huang, Yang Xu, Jing Bian, and Yajie Chen performed the literature search, data collection and statistical analysis. Tingting Huang drafted the manuscript. Zhaohu Chu, Shoucai Zhao, and Lingsong Ma modified the manuscript.

## CONFLICT OF INTEREST

The authors have no conflicts of interest to declare.

### PEER REVIEW

The peer review history for this article is available at https://publons.com/publon/10.1002/brb3.2542

## Data Availability

The data that support the findings of this study are available from the corresponding author upon reasonable request.

## References

[brb32542-bib-0001] Barbanti, P. , Aurilia, C. , Fofi, L. , Egeo, G. , & Ferroni, P. (2017). The role of anti‐CGRP antibodies in the pathophysiology of primary headaches. Neurological Sciences, 38(1), 31–35. 10.1007/s10072-017-2907-8 28527063

[brb32542-bib-0002] Benemei, S. , Nicoletti, P. , Capone, J. G. , & Geppetti, P. (2009). CGRP receptors in the control of pain and inflammation. Current Opinion in Pharmacology, 9(1), 9–14. 10.1016/j.coph.2008.12.007 19157980

[brb32542-bib-0003] Connor, K. M. , Shapiro, R. E. , Diener, H. C. , Lucas, S. , Kost, J. , Fan, X. , Fei, K. , Assaid, C. , Lines, C. , & Ho, T. W. (2009). Randomized, controlled trial of telcagepant for the acute treatment of migraine. Neurology, 73(12), 970–977. 10.1212/WNL.0b013e3181b87942 19770473 PMC2754336

[brb32542-bib-0004] Croop, R. , Goadsby, P. J. , Stock, D. A. , Conway, C. M. , Forshaw, M. , Stock, E. G. , Coric, V. , & Lipton, R. B. (2019). Efficacy, safety, and tolerability of rimegepant orally disintegrating tablet for the acute treatment of migraine: A randomised, phase 3, double‐blind, placebo‐controlled trial. Lancet (London, England), 394(10200), 737–745. 10.1016/S0140-6736(19)31606-X 31311674

[brb32542-bib-0005] Detke, H. C. , Goadsby, P. J. , Wang, S. , Friedman, D. I. , Selzler, K. J. , & Aurora, S. K. (2018). Galcanezumab in chronic migraine: The randomized, double‐blind, placebo‐controlled REGAIN study. Neurology, 91(24), e2211–e2221. 10.1212/WNL.0000000000006640 30446596 PMC6329331

[brb32542-bib-0006] Diener, H. C. , Barbanti, P. , Dahlof C. , Reuter U. , Habeck J. , Podhorna J. (2010). BI 44370 TA, an oral CGRP antagonist for the treatment of acute migraine attacks: Results from a phase II study. Cephalalgia, 31(5), 573–584. 10.1177/0333102410388435 21172952

[brb32542-bib-0007] Dodick, D. W. , Ashina, M. , Brandes, J. L. , Kudrow, D. , Lanteri‐Minet, M. , Osipova, V. , Palmer, K. , Picard, H. , Mikol, D. D. , & Lenz, R. A. (2018). ARISE: A Phase 3 randomized trial of erenumab for episodic migraine. Cephalalgia: An International Journal of Headache, 38(6), 1026–1037. 10.1177/0333102418759786 29471679

[brb32542-bib-0008] Dodick, D. W. , Lipton, R. B. , Ailani, J. , Lu, K. , Finnegan, M. , Trugman, J. M. , & Szegedi, A. (2019). Ubrogepant for the treatment of migraine. The New England Journal of Medicine, 381(23), 2230–2241. 10.1056/NEJMoa1813049 31800988

[brb32542-bib-0009] Dodick, D. W. , Lipton, R. B. , Silberstein, S. , Goadsby, P. J. , Biondi, D. , Hirman, J. , Cady, R. , & Smith, J. (2019). Eptinezumab for prevention of chronic migraine: A randomized phase 2b clinical trial. Cephalalgia: An International Journal of Headache, 39(9), 1075–1085. 10.1177/0333102419858355 31234642

[brb32542-bib-0010] Edvinsson, J. , Grell, A. S. , Warfvinge, K. , Sheykhzade, M. , Edvinsson, L. , & Haanes, K. A. (2020). Differences in pituitary adenylate cyclase‐activating peptide and calcitonin gene‐related peptide release in the trigeminovascular system. Cephalalgia: An International Journal of Headache, 40(12), 1296–1309. 10.1177/0333102420929026 32486909

[brb32542-bib-0011] Edvinsson, L. , Haanes, K. A. , Warfvinge, K. , & Krause, D. N. (2018). CGRP as the target of new migraine therapies—Successful translation from bench to clinic. Nature Reviews. Neurology, 14(6), 338–350. 10.1038/s41582-018-0003-1 29691490

[brb32542-bib-0012] Goadsby, P. J. , Dodick, D. W. , Ailani, J. , Trugman, J. M. , Finnegan, M. , Lakkis H. , Lu K. , & Szegedi, A. (2019). Orally administered atogepant was efficacious, safe, and tolerable for the prevention of migraine: Results from a phase 2b/3 study. Headache, 59(18–19), 1–208. 10.1111/head.13549

[brb32542-bib-0013] Goadsby, P. J. , Reuter, U. , Hallström, Y. , Broessner, G. , Bonner, J. H. , Zhang, F. , Sapra, S. , Picard, H. , Mikol, D. D. , & Lenz, R. A. (2017). A controlled trial of erenumab for episodic migraine. The New England Journal of Medicine, 377(22), 2123–2132. 10.1056/NEJMoa1705848 29171821

[brb32542-bib-0014] Hewitt, D. J. , Aurora, S. K. , Dodick, D. W. , Goadsby, P. J. , Ge, Y. J. , Bachman, R. , Taraborelli, D. , Fan, X. , Assaid, C. , Lines, C. , & Ho, T. W. (2011). Randomized controlled trial of the CGRP receptor antagonist MK‐3207 in the acute treatment of migraine. Cephalalgia: An International Journal of Headache, 31(6), 712–722. 10.1177/0333102411398399 21383045

[brb32542-bib-0015] Hewitt, D. J. , Martin, V. , Lipton, R. B. , Brandes, J. , Ceesay, P. , Gottwald, R. , Schaefer, E. , Lines, C. , & Ho, T. W. (2011). Randomized controlled study of telcagepant plus ibuprofen or acetaminophen in migraine. Headache, 51(4), 533–543. 10.1111/j.1526-4610.2011.01860.x 21457238

[brb32542-bib-0016] Ho, A. P. , Dahlöf, C. G. , Silberstein, S. D. , Saper, J. R. , Ashina, M. , Kost, J. T. , Froman, S. , Leibensperger, H. , Lines, C. R. , & Ho, T. W. (2010). Randomized, controlled trial of telcagepant over four migraine attacks. Cephalalgia: An International Journal of Headache, 30(12), 1443–1457. 10.1177/0333102410370878 20974601

[brb32542-bib-0017] Ho, T. W. , Mannix, L. K. , Fan, X. , Assaid, C. , Furtek, C. , Jones, C. J. , Lines, C. R. , Rapoport, A. M. , & MK‐0974 Protocol 004 study group (2008). Randomized controlled trial of an oral CGRP receptor antagonist, MK‐0974, in acute treatment of migraine. Neurology, 70(16), 1304–1312. 10.1212/01.WNL.0000286940.29755.61 17914062

[brb32542-bib-0018] Jadad, A. R. , Moore, R. A. , Carroll, D. , Jenkinson, C. , Reynolds, D. J. , Gavaghan, D. J. , & McQuay, H. J. (1996). Assessing the quality of reports of randomized clinical trials: Is blinding necessary? Controlled Clinical Trials, 17(1), 1–12. 10.1016/0197-2456(95)00134-4 8721797

[brb32542-bib-0019] Lipton, R. B. , Croop, R. , Stock, E. G. , Stock, D. A. , Morris, B. A. , Frost, M. , Dubowchik, G. M. , Conway, C. M. , Coric, V. , & Goadsby, P. J. (2019). Rimegepant, an oral calcitonin gene‐related peptide receptor antagonist, for migraine. The New England Journal of Medicine, 381(2), 142–149. 10.1056/NEJMoa1811090 31291516

[brb32542-bib-0020] Lipton, R.B. , Dodick, D.W. , Ailani, J. , Lu, K. , Lakkis, H. , Finnegan M. , Szegedi A. , Trugman J.M. (2018). Efficacy, safety, and tolerability of ubrogepant for the acute treatment of migraine: Results from a single attack phase iii study, ACHIEVE II. Headache, 58(8), 1315–1316. 10.1111/head.13411

[brb32542-bib-0021] Lipton, R. B. , Dodick, D. W. , Ailani, J. , Lu, K. , Finnegan, M. , Szegedi, A. , & Trugman, J. M. (2019). Effect of ubrogepant vs placebo on pain and the most bothersome associated symptom in the acute treatment of migraine: The ACHIEVE II randomized clinical trial. JAMA, 322(19), 1887–1898. 10.1001/jama.2019.16711 31742631 PMC6865323

[brb32542-bib-0022] Marcus, R. , Goadsby, P. J. , Dodick, D. , Stock, D. , Manos, G. , & Fischer, T. Z. (2014). BMS‐927711 for the acute treatment of migraine: A double‐blind, randomized, placebo controlled, dose‐ranging trial. Cephalalgia: An International Journal of Headache, 34(2), 114–125. 10.1177/0333102413500727 23965396

[brb32542-bib-0023] Messlinger K. (2018). The big CGRP flood—Sources, sinks and signalling sites in the trigeminovascular system. The Journal of Headache and Pain, 19(1), 22. 10.1186/s10194-018-0848-0 29532195 PMC5847494

[brb32542-bib-0024] Moher, D. , Liberati, A. , Tetzlaff, J. , Altman, D. G. , & PRISMA Group (2009). Preferred reporting items for systematic reviews and meta‐analyses: The PRISMA statement. PLoS Medicine, 6(7), e1000097. 10.1371/journal.pmed.1000097 19621072 PMC2707599

[brb32542-bib-0025] Mulleners, W. M. , Kim, B. K. , Láinez, M. , Lanteri‐Minet, M. , Pozo‐Rosich, P. , Wang, S. , Tockhorn‐Heidenreich, A. , Aurora, S. K. , Nichols, R. M. , Yunes‐Medina, L. , & Detke, H. C. (2020). Safety and efficacy of galcanezumab in patients for whom previous migraine preventive medication from two to four categories had failed (CONQUER): A multicentre, randomised, double‐blind, placebo‐controlled, phase 3b trial. The Lancet. Neurology, 19(10), 814–825. 10.1016/S1474-4422(20)30279-9 32949542

[brb32542-bib-0026] Olesen, J. , Diener, H. C. , Husstedt, I. W. , Goadsby, P. J. , Hall, D. , Meier, U. , Pollentier, S. , Lesko, L. M. , & BIBN 4096 BS Clinical Proof of Concept Study Group (2004). Calcitonin gene‐related peptide receptor antagonist BIBN 4096 BS for the acute treatment of migraine. The New England Journal of Medicine, 350(11), 1104–1110. 10.1056/NEJMoa030505 15014183

[brb32542-bib-0027] Reuter, U. , Goadsby, P. J. , Lanteri‐Minet, M. , Wen, S. , Hours‐Zesiger, P. , Ferrari, M. D. , & Klatt, J. (2018). Efficacy and tolerability of erenumab in patients with episodic migraine in whom two‐to‐four previous preventive treatments were unsuccessful: A randomised, double‐blind, placebo‐controlled, phase 3b study. Lancet (London, England), 392(10161), 2280–2287. 10.1016/S0140-6736(18)32534-0 30360965

[brb32542-bib-0028] Silberstein, S. D. , Dodick, D. W. , Bigal, M. E. , Yeung, P. P. , Goadsby, P. J. , Blankenbiller, T. , Grozinski‐Wolff, M. , Yang, R. , Ma, Y. , & Aycardi, E. (2017). Fremanezumab for the preventive treatment of chronic migraine. The New England Journal of Medicine, 377(22), 2113–2122. 10.1056/NEJMoa1709038 29171818

[brb32542-bib-0029] Skljarevski, V. , Matharu, M. , Millen, B. A. , Ossipov, M. H. , Kim, B. K. , & Yang, J. Y. (2018). Efficacy and safety of galcanezumab for the prevention of episodic migraine: Results of the EVOLVE‐2 Phase 3 randomized controlled clinical trial. Cephalalgia: An International Journal of Headache, 38(8), 1442–1454. 10.1177/0333102418779543 29848108

[brb32542-bib-0030] Stauffer, V. L. , Dodick, D. W. , Zhang, Q. , Carter, J. N. , Ailani, J. , & Conley, R. R. (2018). Evaluation of galcanezumab for the prevention of episodic migraine: The EVOLVE‐1 randomized clinical trial. JAMA Neurology, 75(9), 1080–1088. 10.1001/jamaneurol.2018.1212 29813147 PMC6143119

[brb32542-bib-0031] Sun, H. , Dodick, D. W. , Silberstein, S. , Goadsby, P. J. , Reuter, U. , Ashina, M. , Saper, J. , Cady, R. , Chon, Y. , Dietrich, J. , & Lenz, R. (2016). Safety and efficacy of AMG 334 for prevention of episodic migraine: a randomised, double‐blind, placebo‐controlled, phase 2 trial. The Lancet. Neurology, 15(4), 382–390. 10.1016/S1474-4422(16)00019-3. 10.1016/S1474-4422(16)00019-3. 26879279

[brb32542-bib-0032] Szkutnik‐Fiedler D. (2020). Pharmacokinetics, pharmacodynamics and drug‐drug interactions of new anti‐migraine drugs‐lasmiditan, gepants, and calcitonin‐gene‐related peptide (CGRP) receptor monoclonal antibodies. Pharmaceutics, 12(12), 1180. 10.3390/pharmaceutics12121180 PMC7761673 33287305

[brb32542-bib-0033] Tepper, S. , Ashina, M. , Reuter, U. , Brandes, J. L. , Doležil, D. , Silberstein, S. , Winner, P. , Leonardi, D. , Mikol, D. , & Lenz, R. (2017). Safety and efficacy of erenumab for preventive treatment of chronic migraine: a randomised, double‐blind, placebo‐controlled phase 2 trial. The Lancet. Neurology, 16(6), 425–434. 10.1016/S1474-4422(17)30083-2 28460892

[brb32542-bib-0034] Voss, T. , Lipton, R. B. , Dodick, D. W. , Dupre, N. , Ge, J. Y. , Bachman, R. , Assaid, C. , Aurora, S. K. , & Michelson, D. (2016). A phase IIb randomized, double‐blind, placebo‐controlled trial of ubrogepant for the acute treatment of migraine. Cephalalgia: An International Journal of Headache, 36(9), 887–898. 10.1177/0333102416653233 27269043

